# *ESR1* F404 Mutations and Acquired Resistance to Fulvestrant in *ESR1*-Mutant Breast Cancer

**DOI:** 10.1158/2159-8290.CD-22-1387

**Published:** 2023-11-17

**Authors:** Belinda Kingston, Alex Pearson, Maria Teresa Herrera-Abreu, Li-Xuan Sim, Rosalind J. Cutts, Heena Shah, Laura Moretti, Lucy S. Kilburn, Hannah Johnson, Iain R. Macpherson, Alistair Ring, Judith M. Bliss, Yingwei Hou, Weiyi Toy, John A. Katzenellenbogen, Sarat Chandarlapaty, Nicholas C. Turner

**Affiliations:** 1 The Breast Cancer Now Toby Robins Research Centre, The Institute of Cancer Research, London, United Kingdom.; 2 Clinical Trials and Statistics Unit at The Institute of Cancer Research, London, United Kingdom.; 3 School of Cancer Sciences, University of Glasgow, Glasgow, United Kingdom.; 4 Breast Unit, The Royal Marsden Hospital, London, United Kingdom.; 5 Department of Chemistry and Cancer Center at Illinois, University of Illinois at Urbana-Champaign, Urbana, Illinois.; 6 Memorial Sloan Kettering Cancer Center, New York City, New York.; 7 Department of Medicine, Weill Cornell Medical College, New York City, New York.

## Abstract

Fulvestrant is used to treat patients with hormone receptor–positive advanced breast cancer, but acquired resistance is poorly understood. PlasmaMATCH Cohort A (NCT03182634) investigated the activity of fulvestrant in patients with activating *ESR1* mutations in circulating tumor DNA (ctDNA). Baseline *ESR1* mutations Y537S are associated with poor outcomes and Y537C with good outcomes. Sequencing of baseline and EOT ctDNA samples (*n* = 69) revealed 3/69 (4%) patients acquired novel *ESR1* F404 mutations (F404L, F404I, and F404V), in *cis* with activating mutations. *In silico* modeling revealed that *ESR1* F404 contributes to fulvestrant binding to estrogen receptor–alpha (ERα) through a *pi*-stacking bond, with mutations disrupting this bond. *In vitro* analysis demonstrated that single F404L, E380Q, and D538G models were less sensitive to fulvestrant, whereas compound mutations D538G + F404L and E380Q + F404L were resistant. Several oral ERα degraders were active against compound mutant models. We have identified a resistance mechanism specific to fulvestrant that can be targeted by treatments in clinical development.

**Significance:**

Novel F404 *ESR1* mutations may be acquired to cause overt resistance to fulvestrant when combined with preexisting activating *ESR1* mutations. Novel combinations of mutations in the ER ligand binding domain may cause drug-specific resistance, emphasizing the potential of similar drug-specific mutations to impact the efficacy of oral ER degraders in development.

*This article is featured in Selected Articles from This Issue, p. 201*

## Teaser

As part of Cohort A of the plasmaMATCH trial, baseline ESR1 mutations were found to be predictive of response to fulvestrant, and *ESR1* F404 mutations that occur in cis with classical activating *ESR1* mutations promote resistance.

## INTRODUCTION

For estrogen receptor–positive (ER^+^) breast cancer, which accounts for 75% of breast cancers, hormonal therapy forms the backbone of treatment. In advanced breast cancer (ABC), the selective ER degrader (SERD) fulvestrant is licensed for use in the first and second line, both as a single agent, and in combination with targeted therapies, including CDK4/6 inhibitors and alpelisib (1–3). Fulvestrant acts by competitively inhibiting the binding of estradiol to ERα (4), impeding receptor dimerization and nuclear localization (5, 6), and preventing the activation of estrogen response elements within the regulatory regions of estrogen-sensitive genes. Fulvestrant-bound ER is also unstable, leading to increased degradation of the estrogen receptor (6). Although a standard therapy for patients with ABC, few studies have investigated mechanisms of resistance to fulvestrant.

Activating estrogen receptor mutations (*ESR1* mutations) are acquired through prior aromatase inhibitor therapy for ABC (7), with circulating tumor DNA analysis demonstrating that the mutations are present in 15% to 40% of patients treated with prior aromatase inhibition (8, 9). Activating *ESR1* mutations, which cluster at specific amino acids in the ligand binding domain (LBD), result in ligand-independent activation of *ESR1*. Fulvestrant binding to mutant ERα is partially impaired, with higher concentrations of fulvestrant required to inhibit mutant ERα *in vitro* (5, 10). It is considered unlikely that fulvestrant achieves concentrations required to optimally inhibit mutant *ESR1* in the clinic, and new oral SERDS that do fully inhibit *ESR1*, such as elacestrant, have improved activity as single agents (11–13).

The plasmaMATCH trial investigated the activity of a range of targeted treatments in patients selected based on plasma circulating tumor DNA (ctDNA) testing. Cohort A enrolled patients with ER^+^ ABC with activating *ESR1* mutations for treatment with fulvestrant. Prior clinical research suggests a fulvestrant dose response (14, 15), and patients were treated with extended-dose fulvestrant (500 mg) given every 2 weeks, twice as frequently as standard dosing, to increase fulvestrant exposure and target *ESR1* mutant cancers. Median progression-free survival (PFS) was 2.2 months (16). Here we investigate the genomic associations of response and resistance to fulvestrant in Cohort A of the plasmaMATCH trial. We demonstrate that baseline *ESR1* variants are predictive of response to fulvestrant, with frequent acquisition of potentially targetable mutations. We identify mutations at F404 in estrogen receptor, which occur in *cis* with classic activating *ESR1* mutations, and are acquired as a mechanism of resistance to fulvestrant, identifying the first mechanism of acquired resistance specific to fulvestrant.

## RESULTS

### Baseline *ESR1* Variants and Differential Fulvestrant Activity

**Figure 1.**
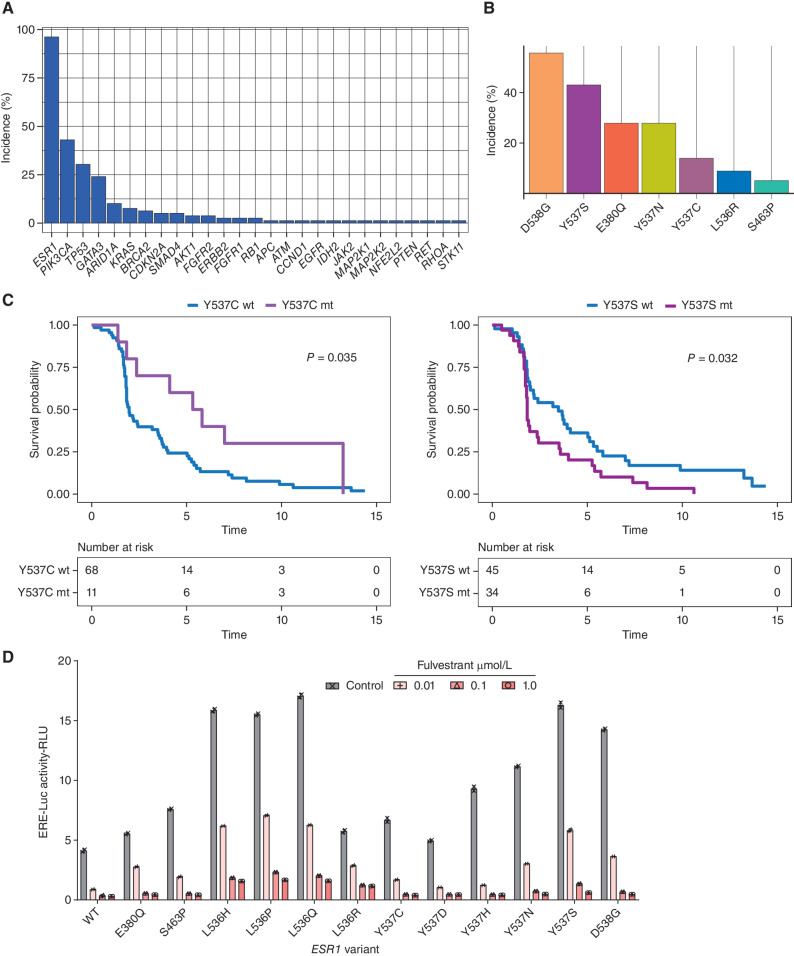
Baseline *ESR1* mutations and fulvestrant efficacy. **A,** % Incidence of mutations in indicated genes at baseline in Cohort A (*n* = 79 assessable patients). **B,** Incidence of baseline *ESR1* alterations within Cohort A (*n* = 79 assessable patients). **C,** PFS of patients in Cohort A, divided by baseline *ESR1* Y537C mutation status (left) and *ESR1* Y537S mutation status (right). *P* values from the log-rank test. HR >1 denotes worse PFS for that group. WT, wild-type; mt, mutant. **D,** MCF7 cells were cotransfected with the indicated *ESR1* expression constructs and treated with the indicated concentration of fulvestrant in the presence of 1 nmol/L estradiol for 24 hours and estrogen response element-luciferase reporter activity determined. Two independent experiments.

Of the 84 patients enrolled in Cohort A treated with extended-dose fulvestrant, 79 (94%) had targeted sequencing results available for analysis, all of whom had detectable ctDNA. The observed baseline mutations reflected the profile of aromatase inhibitor pretreated ABC. Mutations in *ESR1* (96%, 76/79 patients), *PIK3CA* (43% 34/79 patients), and *TP53* (30% 24/79 patients) were the most commonly identified at baseline (Fig. 1A). Median PFS in patients with neither *PIK3CA* nor *TP53* mutations was not significantly altered (Supplementary Fig. S1A and S1B). The most frequent activating *ESR1* alterations in the cohort were D538G (*n* = 44, 55.7%), Y537S (*n* = 34, 43.0%), E380Q (*n* = 22, 27.9%), Y537N (*n* = 22, 27.9%), Y537C (*n* = 11, 13.9%), L536R (*n* = 7, 8.9%), and S463P (*n* = 4, 5.1%; Fig. 1B). We assessed the impact baseline *ESR1* mutations had on fulvestrant efficacy. Patients with detectable baseline Y537C alterations had longer median PFS on fulvestrant compared with patients with other baseline *ESR1* mutations (5.6 months detected versus 2.0 months not detected, HR 2.8; 95% CI, 1.3–5.9; Fig. 1C, left). Conversely, patients with a baseline Y537S mutation had shorter median PFS (1.8 detected vs. 3.5 months not detected, HR 0.53; 95% CI, 0.33–0.86; Fig. 1C, right). Median PFS in patients on fulvestrant with a baseline D538G, E380Q, and Y537N mutations was not significantly different compared with patients with other baseline *ESR1* mutations (Supplementary Fig. S1C–S1E). To assess the impact of common activating mutations on fulvestrant activity *in vitro*, we conducted a screen of MCF7 cells with transient transfection of mutant *ESR1* expression constructs, assessing the impact of mutations on fulvestrant activity on an estrogen response element (ERE) reporter construct. Matching the clinical observations, Y537S induced a high level of resistance to fulvestrant, while Y537C was more sensitive (Fig. 1D). This provides further evidence for fulvestrant resistance of Y537S mutations, adding to the prior data *in vitro* and *in vivo* (10, 17–19), and clinical trial data (20).

### Acquired Mutations on Fulvestrant

**Figure 2.**
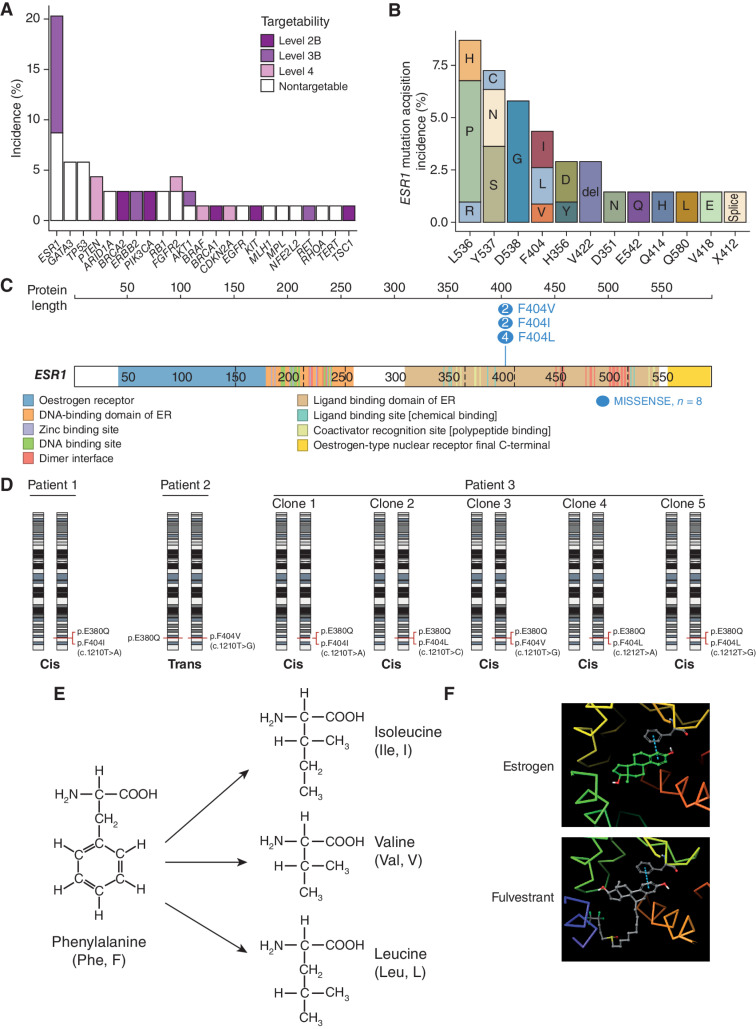
Acquired mutations on fulvestrant. **A,** Incidence of acquired alterations (*n* = 69 assessable patients), colored by targetability of the alterations (Methods). Level 2B denotes the highest level of supporting evidence (“Standard care biomarker recommended by the National Comprehensive Cancer Network or other professional advice guidelines predictive of response to an FDA-approved drug”), whereas level 4 is the lowest (“Compelling biochemical evidence supports the biomarker as being predictive of response to a drug”). **B,** Incidence of acquired *ESR1* mutations (*n* = 14 patients) and resultant amino acid changes. **C,***ESR1* F404 locus in the DNA-binding domain of the estrogen receptor. The number of base changes identified within the data set that result in the three different missense mutations are illustrated using https://proteinpaint.stjude.org/ (36). **D,***cis/trans* analysis of F404 and E380Q in the three patients with assessable targeted sequencing data. Both alleles of chromosome 6 are represented, with annotated locations of the F404 and E380Q on each respective allele representing the *cis/trans* relationship of the variants. **E,** Mutations at phenylalanine 404 result in the substitution of amino acid residues without an aromatic ring. **F,***In silico* modeling predicts the aromatic ring of F404 contributes to a *pi*-stacking bond between the receptor and both estrogen and fulvestrant.

Progression plasma DNA was sequenced in 70 patients, of whom 69 had a baseline plasma sequenced (69/84, 82% enrolled patients). Pathogenic alterations were acquired in 51% of patients (35/69), particularly within estrogen and PI3K/AKT signaling pathways (Fig. 2A; Supplementary Fig. S1F), including 17/69 (25%) patients who acquired potentially targetable alterations, in genes including *PTEN, BRCA1/2, PIK3CA, HER2*, and *BRAF* (Fig. 2A). The total number of acquired alterations was not different in patients who gained clinical benefit (partial response/stable disease ≥24 weeks) versus those who did not (Supplementary Fig. S1G). For *ESR1* mutations, the majority of patients (*n* = 50, 72.5%) maintained their respective poly- or monoclonal *ESR1* mutations, with 5.8% (*n* = 4) acquiring polyclonal disease through the course of treatment. In all, 14/69 (20%) patients acquired *ESR1* mutations at progression, including 6/69 (9%) patients who acquired L536 mutations. This matched the result of our *ESR1* activation mutation ERE screen, in which L536 mutations were the most resistant to fulvestrant (Fig. 1D), likely suggesting that L536 clones were selected through treatment due to fulvestrant resistance.

### Identification and Investigation of *ESR1* F404, a Novel Acquired Mutation

We noted that 3/69 (4%) patients acquired mutations at F404 on progression (Fig. 2B), a mutation that had not previously been described among *ESR1* mutations, including one patient with five separate F404 mutations. The F404 locus is situated within the LBD of *ESR1*, with codon TTT encoding the phenylalanine (Fig. 2C). All three patients had either a partial response or stable disease as their best response on fulvestrant. Of the patients with PFS ≥16 weeks, 12% acquired F404 mutations. We additionally identified H356Y mutations in 3/69 (4%) patients, all in patients with an activating L536P mutation, although subsequent functional experiments suggested that H356Y mutation did not affect ERα function (Supplementary Fig. S2A and S2B).

All three of the patients with acquired F404 mutations harbored activating *ESR1* E380Q mutations at baseline, while two of the patients also had baseline D538G mutations. *Cis/trans* analysis of the three patients with comutant E380Q (a locus close enough to F404 to be able to establish *cis/trans* patterns in ctDNA) revealed that 6/7 F404 base changes detected in these patients occurred in *cis* with the E380Q mutation (Fig. 2D; Supplementary Fig. S3). The patient with the mutation in *trans* with E380Q had additional *ESR1* mutations (D538G, S463P, and Y537N), and it is possible that the F404 mutation was in *cis* with one of those mutations.

In the absence of prior fulvestrant exposure, F404 mutations were very rare. Only 1/800 (0.1%) screening plasma samples from the plasmaMATCH study had an F404 mutation, and this one patient had previously received fulvestrant and had activating mutations in *ESR1* at D538G, E380Q, S463P, and Y537N. Furthermore, we interrogated other ctDNA data sets. In the PIPA combination study of fulvestrant, palbociclib, and taselisib, 1/16 (6%) patients acquired an F404 mutation at progression (21). In the SERENA-1 study of the novel SERD camizestrant, baseline F404 mutations were identified in 2/214 (1%) patients, both of whom had had prior fulvestrant exposure and had other activating *ESR1* mutations (22). Therefore, F404 mutations were found only with prior fulvestrant exposure, only in combination with other classic activating *ESR1* mutations, and occurred in *cis* with activating mutations expected to result in a translated protein that would carry the compound amino acid changes.

The F404 amino acid residue contains an aromatic ring that, when estrogen is bound to the receptor, forms a *pi*-stacking bond with a corresponding aromatic ring within estrogen. Within the patients who harbored an F404 alteration, all base changes lead to the substitution of phenylalanine with one of either isoleucine, valine, or leucine, all of which lack an aromatic ring (Fig. 2E). Fulvestrant has a similar structure to estrogen and includes an aromatic ring that forms a *pi*-staking bond with F404 in structural modeling (Fig. 2F). *In silico* analysis of binding energies (Supplementary Methods), on mutant *ESR1* backgrounds (Y537S or L536S), suggested mutations at F404 reduced the binding affinity of estrogen and fulvestrant to the estrogen receptor (Supplementary Table S1). This potentially explains the clinical observation that F404 mutations only occurred in the presence of other activating *ESR1* mutations, as F404 mutation might otherwise impair estrogen binding and receptor activation in a wild-type ERα receptor.

### Generation and Validation of *ESR1* F404L Models

We investigated the functional consequences of F404 alteration, and the potential role in fulvestrant resistance, using both CRISPR knock-in models and transfection of expression constructs. For both approaches, *ESR1* 1210T>C (F404L), one of the most frequently identified F404 variants, was modeled as a single mutation (F404L) or as a compound mutation in *cis* alongside activating *ESR1* mutations, D538G (1613A>G) and E380Q (1138G>C) selected for investigation as the most frequently co-occurring mutations in the clinical data set.

**Figure 3.**
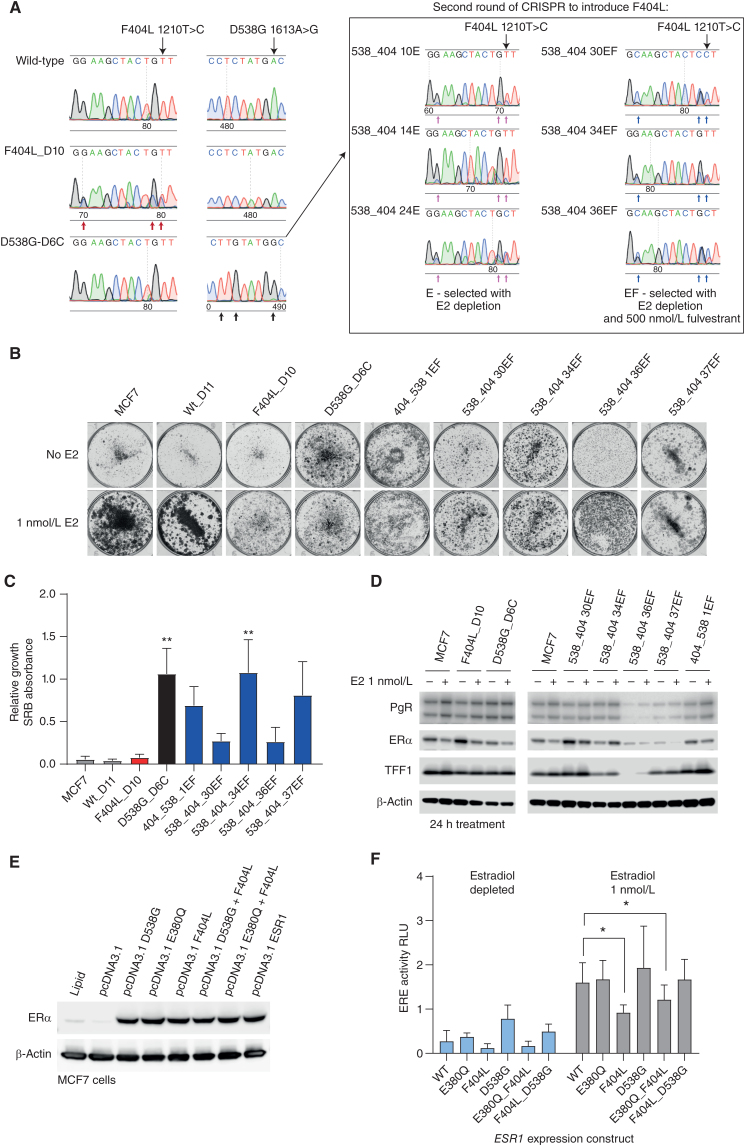
F404 does not activate estrogen signaling. **A,** CRISPR clones of MCF7 cells expressing *ESR1* F404L (1210T>C, CRISPR edit indicated by red arrows) or D538G (1613A>G; CRISPR edit indicated by black arrows) were identified by RT-PCR followed by Sanger sequencing (left-hand panels). Similarly, a second round of CRISPR was used to introduce *ESR1* F404L (1210T>C) into a clone (D6C) that expressed D538G (1613A>G; right-hand panels). **B,** Estrogen-dependent growth was assessed in colony formation assay. Parental MCF7 cells and indicated *ESR1*-mutant models were grown in either the absence or presence of estradiol (1 nmol/L) for 14 days. **C,** Quantification of colony formation assays of *ESR1*-mutant models treated with and without estradiol (1 nmol/L). Sulforhodamine B (SRB)-stained colonies were dissolved, and absorbance at 565 nm was measured. Mean with SEM, *n* = 3 independent experiments, nonparametric one-way ANOVA with Dunn multiple comparisons test; **, *P* < 0.01. **D,** Expression of estrogen target genes, progesterone receptor (PgR), and trefoil factor-1 (TFF1), assessed by western blot in parental MCF7 cells and indicated *ESR1*-mutant models grown in either the absence or presence of estradiol (1 nmol/L) for 24 hours. **E,** MCF7 cells were transfected with *ESR1* expression constructs with indicated *ESR1* variants. Expression of ERα was determined by western blot. **F,** MCF7 cells were cotransfected with the indicated *ESR1* expression constructs ERE-luciferase reporter and control construct. Cells were treated in either the absence or presence of estradiol (1 nmol/L) for 24 hours, and ERE-luciferase activity was assessed. Two-way repeated-measures ANOVA with Dunnett multiple comparisons test, *n* = 4 mean with SD; *, *P* < 0.05. WT, wild-type.

MCF7 cells were subjected to CRISPR-Cas9 with homology-directed repair (HDR) to “knock in” the target mutations. Clones were screened by the Sanger sequencing of genomic DNA. Any clones identified to harbor the targeted mutations were expanded and expression of the mutant transcript was confirmed by RT-PCR and Sanger sequencing (Fig. 3A). Three of 72 (4%) F404 clones harbored the mutation, of which only one of three (33%; F404L_D10) was found to express F404L. Three of 59 (5%) D538G clones harbored the mutation, all of which 3 of 3 (100%) expressed the mutant protein. One of the D538G clones, D538G_D6C, was noted to be homozygous for the mutation providing an ideal background into which to knock in the p.F404L (Fig. 3A). A second round of CRISPR was used to introduce F404L into the D538G_D6C model, with cells divided into pools and subjected to estrogen-free conditions without (E) and with (EF) fulvestrant (0.5 μmol/L). Four of 24 (17%) clones selected in the absence of estrogen (E) had the expression of F404L (Fig. 3A). In contrast, 28 of 30 (93%) clones selected with fulvestrant (EF) had expression of F404L, providing clear evidence of preferential selection.

Growth of both the parental MCF7 and F404L_D10 cells was estrogen dependent. In contrast, all models expressing D538G, and compound D538G + F404L, exhibited estrogen-independent growth (Fig. 3B and C). Similarly, D538G expressing models showed estradiol-independent expression of the estrogen target gene progesterone receptor (PgR) and trefoil factor1 (TFF1; Fig. 3D), whereas F404L showed estradiol-dependent expression. Using an ERE-luciferase reporter gene construct and transient expression, we further assessed the impact of F404L and compound F404L+D538G mutations on estrogen-mediated signaling (Fig. 3E). Cells transfected with D538G tended to increase ERE activity in the absence of estrogen compared with cells expressing wild-type *ESR1* (Fig. 3F). Notably, cells expressing F404L showed lower ERE activity compared with cells expressing wild-type *ESR1* when exposed to estrogen (*P* = 0.0488, *n* = 4; Fig. 3F). Similarly, the combination of E380Q, a less potent activator of ER signaling than D538G, and F404L reduced ERE activity compared with wild-type *ESR1* (*P* < 0.023, *n* = 4). Together, these results are consistent with the hypothesis that F404L affects the LBD of ERα, without activating the receptor.

### Compound F404 Mutations and Resistance to Fulvestrant

**Figure 4.**
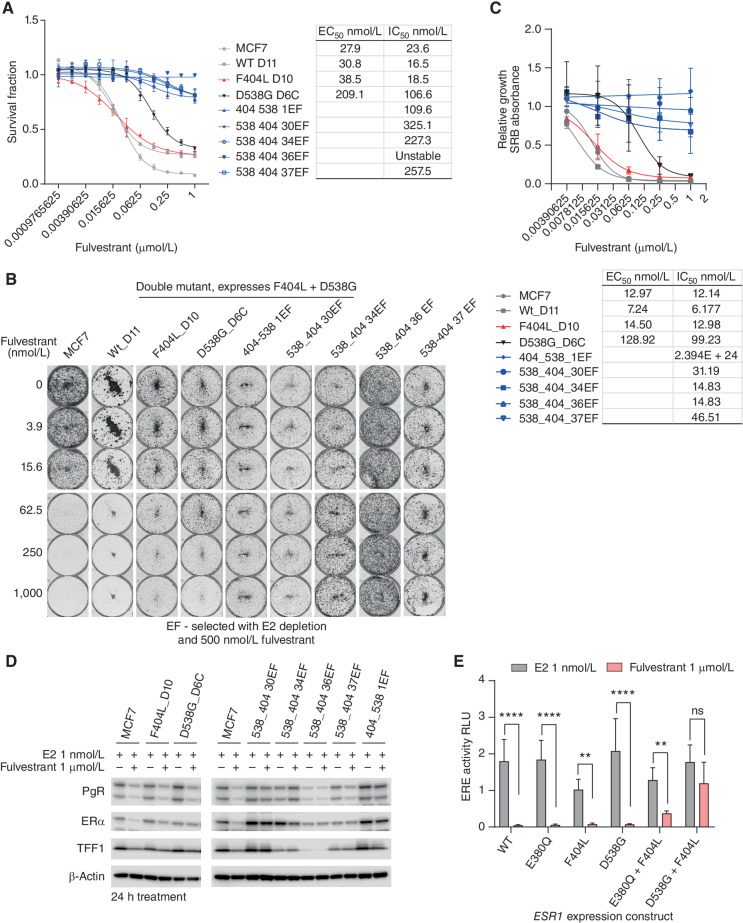
Compound F404L mutations induce resistance to fulvestrant. **A,** Compound mutations of D538G-F404L in MCF7 cells, along with single mutations and wild-type (WT), with sensitivity to fulvestrant assessed after 6 days treatment with Cell-Titer Glo viability assay. *n* = 4 mean with SD. **B,** Representative images of clonongenic assays grown in indicated concentrations of fulvestrant for 14 days. **C,** Quantification of colony formation assays for *ESR1*-mutant models treated with the indicated concentrations of fulvestrant for 14 days. EC_50_ and IC_50_ values were calculated from the response curves. SRB-stained colonies were dissolved, and absorbance at 565 nm was measured. Mean with SEM, *n* = 3 independent experiments. **D,** Expression of estrogen target genes, progesterone receptor (PgR), and trefoil factor-1 (TFF1) was assessed by western blot in parental MCF7 cells and indicated *ESR1*-mutant models grown in the presence of 1 nmol/L estradiol or 1 μmol/L fulvestrant. **E,** MCF7 cells were cotransfected with the indicated *ESR1* expression constructs ERE-luciferase reporter and control construct. Cells were treated with 1 nmol/L estradiol either in the absence or presence of fulvestrant (1 μmol/L) for 24 hours, and ERE-luciferase activity was assessed. Two-way repeated-measures ANOVA with Sidak multiple comparisons test, *n* = 4 mean with SD; **, *P* < 0.01; ***, *P* < 0.001; ****, *P* < 0.0001.

We explored the impact of F404L on the sensitivity of MCF7 cells to fulvestrant. CRISPR models expressing F404L had modestly reduced sensitivity to fulvestrant compared with parental MCF7 cells in both short- and long-term assays (Fig. 4A–C). Resistance to fulvestrant was substantially more marked in compound D538G + F404L models showing profound resistance (Fig. 4A and B). Similarly, quantification of long-term colony formation assays showed the compound D538G + F404L models clear resistance to fulvestrant (Fig. 4C). Single mutant CRISPR F404L, D538G models, and parental MCF7 cells had decreased expression of PgR, TFF1, and ERα when treated with fulvestrant (Fig. 4D). In contrast, models with compound D538G + F404L had limited changes in expression of PgR, TFF1, and ERα when treated with fulvestrant (Fig. 4D). Supporting these observations, ERE activity associated with transient expression of single and compound *ESR1* variants was reduced by treatment with fulvestrant, with the exception of D538G + F404L, which maintained ERE acti­vity compared with cells treated with estradiol alone (Fig. 4E). Consistent with this, the combination of F404L + L536P, a combination not seen in the clinical data set, maintained ERE activity when treated with fulvestrant (Supplementary Fig. S3). Together, these data confirm that the combined effect of compound F404 and activating *ESR1* mutations in *cis* in the same protein caused profound fulvestrant resistance.

### Compound F404 Mutations Increase Estrogen-Dependent Gene Expression

**Figure 5.**
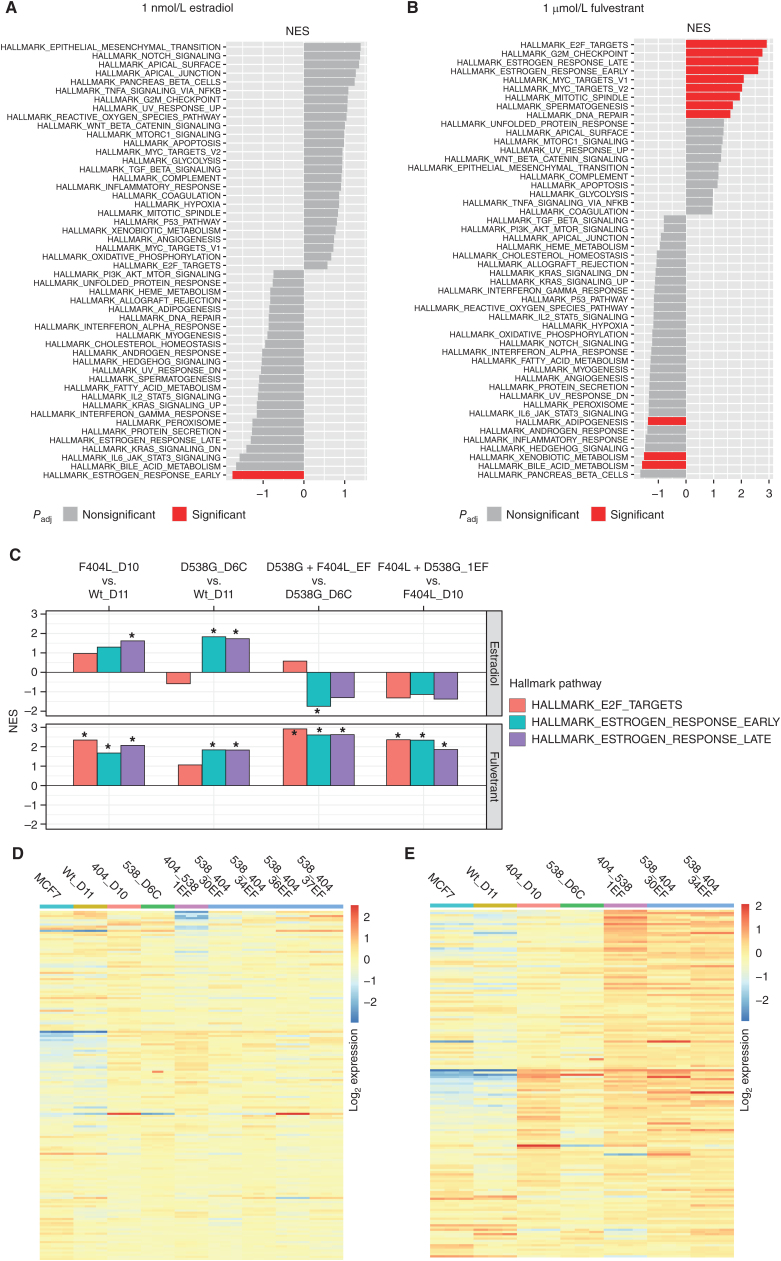
Transcriptomic analysis of *ESR1*-mutant models. **A,** Gene set enrichment analysis (GSEA) for D538G + F404L models compared with D538G D6C cells maintained in 1 nmol/L estradiol. Pathways are highlighted red; false discovery rate-adjusted *q* value <0.05. **B,** GSEA for D538G + F404L models compared with D538G D6C cells treated with 1 μmol/L fulvestrant for 24 hours. Pathways are highlighted red; false discovery rate-adjusted *q* value <0.05. **C,** GSEA for *ESR1*-mutant models. Normalized enrichment score (NES) is shown for the indicated pathways. *, False discovery rate-adjusted *q* value <0.05. **D,** Heat map of “Estrogen response late” genes (log_2_ expression) for *ESR1*-mutant models maintained in 1 nmol/L estradiol. **E,** Heat map of “Estrogen response late” genes (log_2_ expression) for *ESR1*-mutant models treated with 1 μmol/L fulvestrant in the presence of 1 nmol/L estradiol.

To extend the observations of increased estrogen signaling in F404 compound models treated with fulvestrant (Figs. 3C and 4C), RNA sequencing (RNA-seq) was performed for models grown in estradiol (1 nmol/L) with and without fulvestrant (1 μmol/L) for 24 hours (*n* = 3). Gene set enrichment analysis (GSEA) of D538G + F404L compound mutant models grown with estrogen had decreased “Early estrogen pathway” expression but were otherwise similar to D538G-mutant cells (Fig. 5A, FDR-adjusted *q* < 0.05). However, when treated with fulvestrant for 24 hours, E2F transcription, MYC, proliferation, and estrogen-mediated signaling were all significantly increased in the compound mutant model (Fig. 5B, FDR-adjusted *q* < 0.05). The F404L-D10 model had significant upregulation of estrogen signaling compared with the wild-type control (FDR-adjusted *q* < 0.05). Similarly, estrogen signaling was increased in the D538G-D6C model compared with the wild-type control maintained with and without fulvestrant treatment (FDR-adjusted *q* < 0.05; Fig. 5C). Addition of F404L to D538G (D358G + F404L_EF models) showed significant activation of both E2F target and estrogen response (early and late) pathways with fulvestrant treatment (FDR-adjusted *q* < 0.05; Fig. 5C). Differential response of the late estrogen response genes illustrated in Figs. 5D (estradiol; Supplementary Fig. S4A) and 5E (fulvestrant; Supplementary Fig. S4B).

We noted two observations that suggested *ESR1* F404 mutations might be deleterious in the absence of fulvestrant. F404 compound mutations had lower “Early estrogen pathway” expression (Fig. 5A), and introduction of F404 reduced ERE activity compared with wild-type protein in the presence of estrogen (Fig. 3E). Consistent with this, the three double mutants expressing F404L models that were selected in the presence of estrogen “E” (Fig. 3A), all lost the F404L mutation in long-term growth (Supplementary Fig. S5), likely suggesting a subclonal mutation that was outcompeted by the F404F wild-type clone in long-term growth in the absence of fulvestrant.

### Compound F404 Mutations Are Sensitive to Novel SERDs

**Figure 6.**
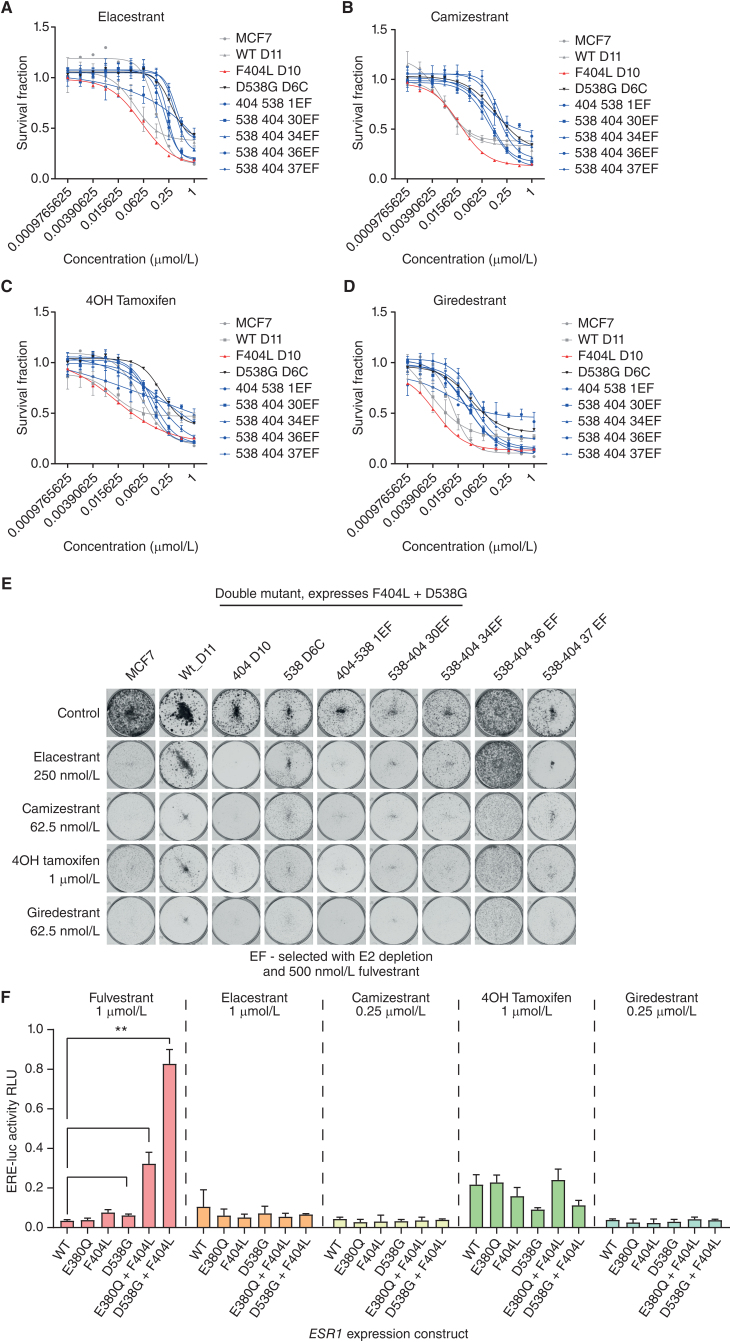
Compound F404 mutations are sensitive to novel SERDs. **A**–**D,** Compound mutations of D538G-F404L in MCF7 cells, along with single mutations and wild-type, with sensitivity to elacestrant (**A**), camizestrant (**B**), 4OH tamoxifen (**C**), and giredestrant (**D**), assessed after 6 days treatment with CellTiter Glo viability assay. *n* = 4 mean with SD. **E,** Representative clonongenic assays grown in indicated concentrations of elacestrant, camizestrant, 4OH tamoxifen, and giredestrant for 14 days. **F,** MCF7 cells were cotransfected with the indicated *ESR1* expression constructs ERE-luciferase reporter and control construct. Cells were treated with indicated concentrations of fulvestrant, elacestrant, camizestrant, 4OH tamoxifen, and giredestrant in the presence of 1 nmol/L estradiol for 24 hours, and ERE-luciferase activity was assessed. Two-way repeated-measures ANOVA with Sidak multiple comparisons test, *n* = 3 mean with SD; *, *P* < 0.05.

*In silico* analysis of binding energies suggested that mutations at F404L may increase the binding affinity of second-generation oral SERDs (Supplementary Table S1). Therefore, we investigated if fulvestrant resistance generated through compound F404 mutations could be overcome by novel SERDs in clinical development, or by the selective estrogen receptor modulator (SERM) tamoxifen. All novel SERDS investigated were active against CRISPR models with both single F404L mutations and D538G + F404L compound mutations, including elacestrant, camizestrant, 4OH tamoxifen, and giredestrant (Fig. 6A–E; Table 1; Supplementary Figs. S6–S9). In particular, models with D538G + F404L compound mutations that were overtly resistant to fulvestrant showed sensitivity to other SERD/SERMs comparable with other D538G expressing models (Fig. 6A–E; Supplementary Figs. S6–S9). Similarly, elacestrant, camizestrant, 4OH tamoxifen, or giredestrant all fully inhibited ERE activity following transient transfection of D538G + F404L and E380Q + F404L (Fig. 6F), despite transfection of these compound mutations resulting in substantial resistance to fulvestrant. Interestingly, 4OH tamoxifen did not completely suppress the acti­vity of the ERE reporter gene assay, with ∼10%–20% activity irrespective of *ESR1* mutation (Fig. 6F), potentially reflecting the difference in mechanism of action between it and the SERDs.

**Table 1.** Calculated IC_50_ and EC_50_ of 4OH tamoxifen and novel SERDs in *ESR1*-mutant models.

		MCF7	WT D11	F404L D10	D538G D6C	404 538 1EF	538 404 30EF	538 404 34EF	538 404 36EF	538 404 37EF
Elacestrant	IC_50_ (nmol/L)	12.2	3.9	5.4	27.7	20.1	23.3	34.6	nc	35.5
	EC_50_ (nmol/L)	16.2	10.7	6.9	59.0	24.9	27.5	46.3	67.0	57.8
Camizestrant	IC_50_ (nmol/L)	1.0	0.7	1.8	14.0	9.6	9.9	15.1	7.5	15.8
	EC_50_ (nmol/L)	2.5	2.2	2.2	28.8	10.9	12.2	20.8	47.2	25.3
4OH Tamoxifen	IC_50_ (nmol/L)	5.6	1.2	1.4	16.8	10.3	8.7	14.4	11.1	20.6
	EC_50_ (nmol/L)	8.9	7.1	3.4	37.9	14.9	11.8	21.7	95.3	46.9
Giredestrant	IC_50_ (nmol/L)	1.2	0.3	0.4	3.1	2.5	2.5	3.7	1.0	4.2
	EC_50_ (nmol/L)	1.3	0.8	0.5	6.3	3.1	3.2	4.6	9.2	6.9

Abbreviation: nc, not calculated.

## DISCUSSION

Here, we present a robust genomic analysis of resistance to fulvestrant in *ESR1-*mutant breast cancer using paired circulating tumor DNA sequencing in patients treated with fulvestrant in the plasmaMATCH study (16). We identify novel *ESR1* mutations that alter F404, that occur only in patients treated with fulvestrant with preexisting activating *ESR1* mutations in their cancer. F404 mutations are acquired in *cis* with a preexisting activating *ESR1* mutation, with the resulting compound mutation resulting in profound resistance to fulvestrant, but with retained sensitivity to a range of novel SERDs, identifying a treatment strategy to overcome acquired resistance conveyed by F404 mutations.

Mutations at F404 do not appear to occur in the absence of fulvestrant exposure, and then only in the presence of other activating *ESR1* mutations. F404 has previously been predicted to form *pi*-stacking bonds with plant polyphenols identified in a screen of compounds as candidates with antiestrogenic properties (23). Similarly, structural analysis suggested that F404 forms a *pi*-stacking bond with an aromatic ring in both estradiol and fulvestrant. Consistent with these predictions, *in vitro*, the introduction of F404 mutations resulted in lower levels of ERE activity compared with wild-type *ESR1* (Fig. 3). Mutation of F404 would likely reduce *ESR1* activity in the absence of other *ESR1* mutations, which may have a deleterious effect on tumor growth, explaining the lack of F404 mutations observed without prior acquisition of an activating *ESR1* mutation. Compound F404 mutations resulted in profound resistance to fulvestrant, with single F404-mutant models showing more limited fulvestrant resistance. It is likely that the effect of *ESR1*-activating mutations on the ligand binding pocket, combined with the loss of the *pi*-stacking bond, results in an impairment of fulvestrant affinity for the ligand binding pocket. *In silico* analysis of binding energies was consistent with this hypothesis, although formal *in vitro* studies in the future would be required to assess this (Supplementary Table S1), with the alternative hypothesis being that F404X mutations do not affect the binding of fulvestrant, but affect the conformational change induced by fulvestrant binding. Interesting *in silico* analysis predicted that binding energies of novel SERDs were not affected by, or even promoted by, F404 mutations, and consistent with this the efficacy of novel SERDs, was unaffected by mutations in F404, providing a therapeutic option to circumvent this mechanism of resistance. Investigation of a wider range of SERDs/SERMs is required to confirm whether this resistance mutation is, as is currently suggested, specific to fulvestrant. This endocrine therapy resistance mechanism is unique in leading to reactivation of the estrogen receptor itself, in contrast to other mechanisms such as inactivating *NF1* and *ARID1A* mutations (24, 25), emphasizing the need to identify whether further drug-specific mutations may limit the efficacy of oral ER degraders in clinical development.

Interestingly, our results predict that although F404 compound mutations promote growth in the presence of fulvestrant, this conditional advantage may come at the cost of reduced fitness in the absence of fulvestrant, as F404 mutations may reduce ER signaling in the absence of fulvestrant and therefore come at the cost of impaired clonal growth once fulvestrant is withdrawn (Supplementary Fig. S5). This suggests that for patients with resistance to fulvestrant generated by F404 mutations, there may be the possibility of rechallenging with fulvestrant after a treatment break, as has been seen rechallenging with cetuximab in patients who *KRAS* mutations in colorectal cancer (26).

Our study emphasizes the extent to which tumor genomes may evolve through fulvestrant therapy, with 25% of patients acquiring a potentially targetable driver mutation. Evidence suggests that ER-positive breast cancers may become substantially heterogeneous after progression on endocrine therapy and that heterogeneity presents a considerable challenge to subsequent treatment efficacy (20, 27, 28). The high incidence of mutation “acquisition” was largely driven by the gain of *ESR1* mutations, and likely reflects clonal selection in cancer, while emphasizing the importance of ctDNA liquid biopsy testing to match treatment to current genomics (16). This heterogeneity may be more marked in *ESR1*-mutant cancer, as *ESR1* mutations may co-occur with other mechanisms of genetic resistance, potentially reflecting cancers that are predisposed to acquiring genetic mechanisms of resistance (20, 28) Recently, the acquisition of secondary mutations in *cis* with hotspot driver mutations in *PIK3CA* were described (29), leading to increased signaling and tumor growth. *PIK3CA* double mutants were found to have increased sensitivity to PI3K inhibitors (29). Similarly, we report double mutations in *ESR1* where the primary mutation has been widely described (10, 18, 20, 28, 30), acquired in response to exposure to aromatase inhibitors (7). In contrast to *PIK3CA* double mutations that enhance PI3K signaling, the acquisition of F404 only provides a growth advantage in the context of exposure to fulvestrant.

In conclusion, we identify a novel *ESR1* mutation at ERα F404, that when acquired in combination with an activating *ESR1* mutation induces resistance to the widely used SERD fulvestrant. Mutations at this codon result in changes at F404 to amino acid residues that lack an aromatic ring, disrupting the *pi*-stacking bond with both estradiol and fulvestrant. The resistance of F404 double mutants is specific to fulvestrant and can be overcome by the use of alternate SERDs, suggesting a route to overcome therapeutic resistance in the clinic. Mutations in the estrogen receptor can confer resistance to ER-binding drugs, without promoting ER activity, identifying a new mechanism through which the cancer can become resistant to hormonal therapies.

## METHODS

### Patient Enrollment into plasmaMATCH and Blood Sampling

The plasmaMATCH trial (NCT03182634) was co-sponsored by the Institute of Cancer Research and the Royal Marsden National Health Service (NHS) Foundation Trust, London, UK, and approved by a Research Ethics Committee (16/SC/0271), as previously reported (16). Baseline ctDNA testing was conducted with droplet digital PCR (ddPCR), and from partway through the trial with targeted sequencing in parallel to ddPCR. For patients enrolled prior to prospective targeted sequencing, a banked pretreatment plasma sample was retrospectively sequenced. An additional plasma sample taken at disease progression was also subject to targeted sequencing.

For the baseline ctDNA test, 30 to 40 mL of blood was collected in 3 to 4 10 mL cell-free DNA BCT Streck tubes. 30 mL of blood was shipped at ambient temperature to a central laboratory (Centre for Molecular Pathology, Royal Marsden Hospital) for ddPCR testing and retrospective targeted sequencing. In addition, from partway through the trial 10 mL blood was shipped to Guardant Health for targeted sequencing. An additional sample was collected at cycle 1 day 1, and end-of-treatment sample in 2 × 10 mL BD Vacutainer EDTA tubes, centrifuged within 1 hour of collection, for retrospective targeted sequencing.

### Computer Modeling of Estrogen *Pi*-Stacking with ER

Models of estrogen ligand A-ring *pi*-stacking with F404 in the ligand binding pocket of ERα were generated as follows: There is no crystal structure for fulvestrant bound to ERα; the only related crystal structure is for ICI 164,384, a close fulvestrant analogue, in the other ER subtype, ERβ (PDB ID 1HJ1). Therefore, we removed the ICI 164,384 ligand from this structure, modified the side chain to match that of fulvestrant, and modeled it into the ERα crystal structure for the antiestrogen Bazedoxifene after removing the Bazedoxifene ligand (PDB ID 6PSJ); the fitting was done using Schrödinger Glide (https://www.schrodinger.com/products/glide). The estradiol structure in ERα is from PDB ID 3UUD.

### ctDNA Testing and Analysis

ctDNA targeted sequencing was conducted with Guardant360 that identifies single-nucleotide variants, indels, copy-number alterations, and fusions within protein-coding regions of 73 (version 2.10) or 74 genes (version 2.11), as previously described (28, 31).

Variants from Guardant 360 were annotated with VEP version 96 (32). Germline calls were identified by Guardant360 with additional calls (identified based on a combination of VAF frequency around 50% ± 2% and VAF in the general population in the Genome Aggregation Database >0.001%) excluded. To identify pathogenic mutations, variants were annotated with OncoKB (33) and CancerHotspots (34). Mutations were classified as pathogenic based on Cancer Hotspots or OncoKB annotations or recurrent mutations in key breast cancer genes (*ESR1*, *HER2*, *PIK3CA*, *EGFR*, *RB1*, and *FGFR2*) or splicing mutations. All analyses presented are based on mutations assessed as likely pathogenic. Targetability was assigned using OncoKB annotation, a manually curated database of alterations (33).

### Cell Lines

MCF7 cell lines were obtained from ATCC and cultured in phenol-free RPMI media (32404-014, Life Technologies) supplemented with 10% dextran/charcoal-stripped FBS (12676029, Life Technologies), 1 nmol/L oestradiol (Sigma), glutamine (25030149, Life Technologies), penicillin and streptomycin (15140-122, Life Technologies). Cell lines were banked in multiple aliquots on receipt to reduce the risk of phenotypic drift and identity confirmed by STR profiling with the PowerPlex 1.2 System (Promega). Cell cultures were routinely tested for the presence of *Mycoplasma* using the MycoAlert Detection kit (LT07-318 Lonza).

### Antibodies and Drugs

Antibodies used were ERα (sc543, Santa Cruz Biotechnology), PGR (8757, Cell Signaling Technology), TFF1 (15571, Cell Signaling Technology), and β-actin (A5441 Sigma). Secondary antibodies used were α-rabbit-HRP (7074) and α-mouse-HRP (7076, Cell Signaling Technology). Fulvestrant (S1191), 4OH-tamoxifen (S7827), and camizestrant (S8958) were obtained from Selleck Chemicals. Elacestrant (HY-19822A) and giredestrant (HY-109176) were obtained from MedChemExpress.

### Generation and Analysis of *ESR1*-Mutant CRISPR Models

MCF7 cells were subjected to CRISPR-Cas9 genome editing with HDR using Integrated DNA Technologies (IDT) Alt-R CRISPR-Cas9 system according to the manufacturer's guidelines. Briefly, the day before transfection 250,000 cells were plated per well of a 6-well plate in antibiotic-free media containing HDR enhancer V2 (2 μmol/L, 10007910 IDT). crRNA and HDR templates were designed using IDT's Alt-R CRISPR HRD design tool (https://eu.idtdna.com/pages/tools/alt-r-crispr-hdr-design-tool; Supplementary Table S2). gRNA complexes (1 μmol/L) were prepared by hybridization of targeting crRNA with tracrRNA-ATTO555 (1075928, IDT). Ribonucleoprotein (RNP) complexes were prepared by addition of gRNA complexes, Cas9 (1081060 IDT), HDR template, Cas9 PLUS reagent (Thermo Fisher Scientific), and OptiMEM (31985062, Thermo Fisher Scientific), and incubated for 5 minutes at room temperature. Transfection mixes were prepared using RNP complexes with Lipofectamine CRISPMAX (CMAX00008, Thermo Fisher Scientific) and incubated for 20 minutes at room temperature. Transfection mixes were added to preseeded cells in 6-well plates and incubated overnight. Forty-eight hours after transfection cells were spilled into 10-cm dishes and cells cultured until colonies had established. gDNA was extracted from the transfection pool using QuickExtract DNA Extraction Solution (QE09050 Lucigen), and CRISPR editing was assessed using Alt-R Genome Editing Detection kit (1075932 IDT). After approximately 2 weeks individual colonies were picked into 96-well plates and expanded. gDNA was extracted from colonies using QuickExtract DNA Extraction Solution (QE09050 Lucigen), subjected to PCR (primer details in Supplementary Table S1), PCR products isolated (QIAquick PCR purification kit, 28104 Qiagen) and screened for presence of targeted mutations by Sanger sequencing (Azenta Life Sciences). Clones in which targeted mutations were identified were expanded.

To confirm mutant *ESR1* variants were expressed by selected clones, RNA was extracted using RNeasy Mini Kit (74104, Qiagen), cDNA prepared using SuperScript IV first-strand synthesis kit (18091050, Thermo Fisher Scientific), and amplified using AllTaq PCR Core Kit (203123, Qiagen; primer details in Supplementary Table S1). As described, PCR products were isolated and screened for the presence of targeted mutations by Sanger sequencing (Azenta Life Sciences).

### Fulvestrant Screen of *ESR1*-Mutant Expressing MCF7 Cells

A series of expression constructs with *ESR1* point mutations was generated in the pcDNA3.1 HA-ERα (17). Transfections of MCF7 cells using HA-tagged wild-type or mutant ERα, with 3 × -ERE-TATA-Luciferase reporter and pRL-TK-Renilla luciferase plasmid (Promega) using Lipofectamine 2000 (Life Technologies) were done according to the methods of Toy and colleagues (17). Cells were exposed to fulvestrant at indicated concentrations 1 day after transfection for 24 hours, and luciferase activities were determined using the Dual Luciferase Reporter Assay System (E2920, Promega) according to the manufacturer's instructions. Luciferase bioluminescence measurements were performed with the Veritas Microplate Luminometer (Promega).

### ERE Assays with Transient Transfection

pcDNA3.1+/C-DYK plasmids, with the open reading frame of *ESR1* (NM_000125.4) with and without point mutations (estrogen receptor constructs, ERCs; Supplementary Table S2), were purchased from GenScript (The Netherlands). Sanger sequencing was used to confirm the presence of the desired mutations within the custom insert. MCF-7 cells were seeded in 6-well plates with 250,000 cells per well in antibiotic-free media, the following day transfected using Fugene 6 (Promega) with the ERC, a plasmid expressing an estrogen response element with firefly luciferase (ERE-luciferase; ref. 35) and pRL-CMV (*Renilla* luciferase control, Promega). 24 hours after transfection, experimental conditions were applied for a further 24 hours, and firefly luciferase (ERE activity) and *Renilla* luciferase using the Dual-Glo Luciferase Assay System (E2920, Promega) following the manufacturer's instructions measured with a VICTOR X3 MultiLab. Experiments were repeated a minimum of 3 times.

### *In Vitro* Viability Assessment

Colony formation assays were conducted in 6-well plates, seeded with 10,000 cells/well prior to exposure to the indicated experimental conditions. Plates were fixed with tricyclic acid (10%^v^/_v_), stained with sulforhodamine B (SRB; S1402, Sigma; 0.37% ^w^/_v_, in 1% acetic acid), and colonies were counted using a GelCOUNT instrument (Oxford Technologies). For short-term survival assays, 700 cells/well were plated in 384-well plates and exposed to indicated drugs. Survival was assessed after 6 days of treatment using the CellTiter-Glo cell viability assay (G7572, Promega).

### Western Blotting

Cells were lysed in NP40 lysis buffer (1% v/v NP40, 10 mmol/L Tris–Cl pH8, 150 mmol/L NaCl, 1 mmol/L EDTA, 1 mmol/L DTT) supplemented with protease/phosphatase inhibitor cocktail (5872, Cell Signaling Technologies). Western blots were carried out with precast Bis-Tris gels (Life Technologies).

### RNA-Seq Expression Analysis

*ESR1*-mutant models and controls were treated with 1 nmol/L estradiol ± 1 μmol/L fulvestrant for 24 hours (9 models with estradiol treatment, 7 of which also had fulvestrant treatment, *n* = 3), cells harvested, and RNA extracted using RNeasy Mini Kit (74104, Qiagen). Each cell model was treated in 3 independent experiments.

Forty-eight total RNA samples were sent to Novogene (UK) Company Ltd and subjected to Eukaryotic mRNA-Seq (Illumina Novaseq PE150, Q30 ≥80%). Sequencing data for 48 RNA samples for 9 models using bcbio-nextgen,1.2.4 pipeline, reads were aligned using STAR with version STAR 2.6.1d, counted using salmon, 1.4.0. The data were divided into two parts with respect to treatment with 1 nmol/L estradiol and 1 μmol/L fulvestrant as EST and FUL. The data were normalized using DEseq2 version “1.38.3.” DESeq2 was also used to determine differentially expressed genes between different models of single mutants (404_D10, 538_D6C) versus control (MCF7), single mutants (404_D10, 538_D6C) versus wt_D11 and double mutants (538_404, 404_538) versus single mutants (538_D6C, 404_D10) using shrunken log_2_ fold changes in EST and FUL data, respectively. Heatmaps were generated using pheatmap package version “1.0.12” and ggplots “3.4.2” R package. GSEA was carried out using Molecular Signatures Database “Hallmarks” gene set collection using package fgsea “1.24.0” and clusterProfiler “4.6.2” R packages.

### Statistical Analyses

Statistical analysis was carried out using R version 4.0.5 and GraphPad Prism v8.4.3. Time-to-event survival data were analyzed with a log-rank test, and hazard ratios were calculated with Cox regression. Plots were created using GraphPad Prism v8.4.3 and the R software packages ggplot2 and survminer.

### Data Availability Statement

The processed plasmaMATCH Guardant360 sequencing data generated and analyzed during the current study are available as part of Kingston and colleagues (28). We do not have permission from the patients to publicly deposit the raw sequencing data. To protect the privacy and confidentiality of patients in this study, clinical data are also not made publicly available. The data can be obtained by submitting a formal data access request in accordance with the Institute of Cancer Research Clinical Trials and Statistics Unit (ICR-CTSU) data and sample access policy. Requests are to be made via a standard proforma describing the nature of the proposed research and the extent of data requirements which is reviewed by the trial management group. Data recipients are required to enter a formal data-sharing agreement, which describes the conditions for data release and requirements for data transfer, storage, archiving, publication, and intellectual property. Trial documentation including the protocol is available on request by contacting plasmamatch-icrctsu@icr.ac.uk.

## Supplementary Material

Supplementary Methods Supplementary methods: Computer modeling of SERDs pi-stacking with ER

Supplementary Tables 1-2 Supplementary Table 1. Comparison of Potential Binding Energy and Distance of Pi-Pi Stacking Interaction with F404 and mutant modes. Supplementary Table 2. Clinicopathological features of PlasmaMATCH Cohort A.

Supplementary Figures 1-9 Supplementary Figure 1. Progression free survival of patients with selected mutations. Supplementary Figure 2. H356Y does not activate estrogen signalling or alter fulvestrant sensitivity in combination with L536P. Supplementary Figure 3. Acquired ESR1 F404 mutations in patients treated with Fulvestrant. Supplementary Figure 4. Expression of individual ER target genes in RNAseq experiment. Supplementary Figure 5. Loss of F404L mutation in long-term culture of “E” mutant clones. Supplementary Figure 6. Response of D538G+F404L mutant models to elacestrant. Supplementary Figure 7. Response of D538G+F404L mutant models to camizestrant. Supplementary Figure 8. Response of D538G+F404L mutant models to 4OH tamoxifen. Supplementary Figure 9. Response of D538G+F404L mutant models to giredestrant.
