# Supplementary material for: ESR1 F404 Mutations and Acquired Resistance to Fulvestrant in ESR1-Mutant Breast Cancer
Source: Cancer Discov. 2023 Nov 17;14(2):274–89. doi: 10.1158/2159-8290.CD-22-1387 (PMC10850945; doi:10.1158/2159-8290.CD-22-1387)
Supplement: Supplementary Methods — Computer modeling of SERDs pi-stacking with ER [file cd-22-1387_supplementary_methods_suppsm.docx]

# Title: *ESR1* F404 mutations and acquired resistance to fulvestrant in *ESR1* mutant breast cancer.

## Authors and affiliations:

Belinda Kingston^1^, Alex Pearson^1^, Maria Teresa Herrera-Abreu^1^, Li-Xuan Sim^1^, Rosalind J Cutts^1^, Heena Shah^1^, Laura Moretti^2^, Lucy S Kilburn^2^, Hannah Johnson^2^, Iain R Macpherson^3^, Alistair Ring^4^, Judith M Bliss^2^, Yingwei Hou ^5^, Weiyi Toy^6^, John A Katzenellenbogen^5^, Sarat Chandarlapaty^6^, Nicholas C Turner^1,4^

^1^ The Breast Cancer Now Toby Robins Research Centre, The Institute of Cancer Research, London, SW3 6JB.

^2^ Clinical Trials and Statistics Unit at The Institute of Cancer Research, London, UK

^3^ School of Cancer Sciences, University of Glasgow, Glasgow, G61 1QH

^4^ Breast Unit, The Royal Marsden Hospital, Fulham Road, London.

^5^ Department of Chemistry and Cancer Center at Illinois, University of Illinois at Urbana-Champaign, Urbana, Illinois.

^6^ Memorial Sloan Kettering Cancer Center, New York City; Department of Medicine, Weill Cornell Medical College, New York City, USA.

## Running title:

Mutations of *ESR1* at F404 confer fulvestrant resistance.

## Keywords:

Fulvestrant, acquired resistance, breast cancer.

# Supplementary Methods

## Computer modeling of SERDs pi-stacking with ER

Docking of Fulvestrant with Bazedoxifene-ERα complex (Y537S mutant, PDB ID: 6PSJ) in Schrodinger: Download PDB file of Bazedoxifene- ERα complex from Protein Data Bank (<https://www.rcsb.org/>), The protein was prepared with Schrodinger Protein Preparation Wizard module. Fulvestrant structure was prepared with Ligprep module. Grid of receptor (6PSJ Chain B) was generated with Receptor Grid Generation module with H-bond constraint between E353 and Hydroxyl of A-ring. Docking Fulvestrant in the receptor grid with H-bond constrain and standard precision. Posture of Fulvestrant- ERα (Y537S) complex was obtained.

Preparation of mutant modes: PDB files of Estradiol (PDB ID: 3UUD, Y537S), Elacestrant (PDB ID: 7TE7, L536S), Camizestrant analog (PDB ID: 6ZOR, L536S) and Giredestrant (PDB ID: 7MSA, L372S/L536S) were downloaded from Protein Data Bank, and prepared with Schrodinger Protein Preparation Wizard module. Chain A of 3UUD, Chain B of 7TE7, Chain B of 6ZOR and Chain C of 7MSA were selected based on the integrality of protein and ligands. Using Mutate Residues module in Schrodinger to convert F404 to F404L, F404I and F404V. Refinement of these mutant modes with Refine Protein-Ligand Complex module of Schrodinger within 7.0 Å distance from the ligands.

Calculation of Potential Binding Energy: Split refined complex with ligands and receptors. Calculating potential binding energy with MM-GBSA module of Schrodinger, using take complexes from separated ligand and protein option, and setup flexible residue distance from ligand as 6.0 Å. The potential binding energy (MMGBSA dG Bind) of complexes were obtained.
