# Supplementary material for: ESR1 F404 Mutations and Acquired Resistance to Fulvestrant in ESR1-Mutant Breast Cancer
Source: Cancer Discov. 2023 Nov 17;14(2):274–89. doi: 10.1158/2159-8290.CD-22-1387 (PMC10850945; doi:10.1158/2159-8290.CD-22-1387)
Supplement: Supplementary Tables 1-2 — Supplementary Table 1. Comparison of Potential Binding Energy and Distance of Pi-Pi Stacking Interaction with F404 and mutant modes. Supplementary Table 2. Clinicopathological features of PlasmaMATCH Cohort A. [file cd-22-1387_supplementary_tables_1-2_suppst1-st2.docx]

# Title: *ESR1* F404 mutations and acquired resistance to fulvestrant in *ESR1* mutant breast cancer.

## Authors and affiliations:

Belinda Kingston^1^, Alex Pearson^1^, Maria Teresa Herrera-Abreu^1^, Li-Xuan Sim^1^, Rosalind J Cutts^1^, Heena Shah^1^, Laura Moretti^2^, Lucy S Kilburn^2^, Hannah Johnson^2^, Iain R Macpherson^3^, Alistair Ring^4^, Judith M Bliss^2^, Yingwei Hou ^5^, Weiyi Toy^6^, John A Katzenellenbogen^5^, Sarat Chandarlapaty^6^, Nicholas C Turner^1,4^

^1^ The Breast Cancer Now Toby Robins Research Centre, The Institute of Cancer Research, London, SW3 6JB.

^2^ Clinical Trials and Statistics Unit at The Institute of Cancer Research, London, UK

^3^ School of Cancer Sciences, University of Glasgow, Glasgow, G61 1QH

^4^ Breast Unit, The Royal Marsden Hospital, Fulham Road, London.

^5^ Department of Chemistry and Cancer Center at Illinois, University of Illinois at Urbana-Champaign, Urbana, Illinois.

^6^ Memorial Sloan Kettering Cancer Center, New York City; Department of Medicine, Weill Cornell Medical College, New York City, USA.

## Running title:

Mutations of *ESR1* at F404 confer fulvestrant resistance.

## Keywords:

Fulvestrant, acquired resistance, breast cancer.

# Supplementary Table 1. Comparison of Potential Binding Energy and Distance of Pi-Pi Stacking Interaction with F404 and mutant modes.

| **PDB ID^a^** | **Ligand Structure** | **Binding Free Energy with ER F404** | **Binding Free Energy with ER F404L** | **Binding Free Energy with ER F404I** | **Binding Free Energy with ER F404V** |
| --- | --- | --- | --- | --- | --- |
| **3UUD** (Y537S) |  | -75.31 kcal/mol | -68.09 kcal/mol | -65.90 kcal/mol | -66.28 kcal/mol |
| **Docking** (Y537S) |  | -95.58 kcal/mol | -92.49 kcal/mol | -92.23 kcal/mol | -93.18 kcal/mol |
| **7TE7** (L536S) |  | -108.75 kcal/mol | ***-110.01 kcal/mol*** | -105.90 kcal/mol | -98.33 kcal/mol |
| **6ZOR**  (L536S) |  | -69.40 kcal/mol | ***-74.31 kcal/mol*** | ***-73.82 kcal/mol*** | ***-72.93 kcal/mol*** |
| **7MSA**  (L372S  /L536S) |  | -81.36 kcal/mol | ***-82.42 kcal/mol*** | ***-85.77 kcal/mol*** | -80.98 kcal/mol |

**^a^** Numbers in parentheses are the location of mutations elsewhere in the ER LBD needed to assist in crystallization.

# Supplementary Table 2. Clinicopathological features of PlasmaMATCH Cohort A.

|  | n=84 | |
| --- | --- | --- |
|  | n | % |
| **Age group (years) at registration** |  |  |
| <50 | 18 | 21.4 |
| 50-59 | 36 | 42.9 |
| 60-69 | 20 | 23.8 |
| >=70 | 10 | 11.9 |
|  |  |  |
| **Metastatic disease present at diagnosis** | 18 | 21.4 |
|  |  |  |
| **Time since primary diagnosis (years)** |  |  |
| <1 year | 2 | 2.4 |
| 1-3 years | 11 | 13.1 |
| 3-5 years | 17 | 20.2 |
| >=5 years | 54 | 64.3 |
|  |  |  |
| **Tumour characteristics at initial diagnosis** |  |  |
| Pathological invasive tumor size (cm) |  |  |
| <=2cm | 18 | 21.4 |
| 2-5 cm | 29 | 34.5 |
| >5cm | 11 | 13.1 |
| Not known/Missing | 26 | 31.0 |
|  |  |  |
| **Nodal status** |  |  |
| N0 | 18 | 21.4 |
| N1-3 | 24 | 28.6 |
| N4+ | 20 | 23.8 |
| Not known/Missing | 22 | 26.2 |
|  |  |  |
| **Histological type** |  |  |
| Ductal | 63 | 75.0 |
| Lobular | 9 | 10.7 |
| Mixed ductal and lobular | 5 | 6.0 |
| Other invasive | 1 | 1.2 |
| DCIS | 1 | 1.2 |
| Not known/Missing | 5 | 6.0 |
|  |  |  |
| **Tumor grade** |  |  |
| G1 | 7 | 8.3 |
| G2 | 37 | 44.0 |
| G3 | 28 | 33.3 |
| Not known/Missing | 12 | 14.3 |
|  |  |  |
| **Molecular subtype** |  |  |
| HR+, HER2- | 80 | 95.2 |
| HR+, HER2+ | 3 | 3.6 |
| HR+, HER2 Unknown | 1 | 1.2 |
|  |  |  |
| **Disease sites** |  |  |
| Visceral | 78 | 92.9 |
| Soft tissue/nodal | 6 | 7.1 |
|  |  |  |
| **Treatment received for locally advanced/metastatic disease prior to study registration** |  |  |
|  |  |  |
| **Chemotherap**y |  |  |
| 0 lines | 29 | 34.5 |
| 1 line | 26 | 31.0 |
| 2 lines | 13 | 15.5 |
| >2 lines | 16 | 19.0 |
|  |  |  |
| **Endocrine therapy** |  |  |
| 0 lines | 5 | 6.0 |
| 1 line | 34 | 40.5 |
| 2 lines | 36 | 42.9 |
| 3 lines | 8 | 9.5 |
| 4 lines | 1 | 1.2 |
|  |  |  |
| **Total lines of treatment received (chemotherapy and endocrine therapy combined)** |  |  |
| 0 | 2 | 2.4 |
| 1 | 15 | 17.9 |
| 2 | 23 | 27.4 |
| 3 | 16 | 19.0 |
| 4 | 12 | 14.3 |
| 5 | 11 | 13.1 |
| >5 | 5 | 6.0 |
|  |  |  |
| **Other systemic therapy** |  |  |
| Anti-HER2 therapy | 3 | 3.6 |
| mTOR inhibitor (everolimus, vistusertib) | 18 | 21.4 |
| CDK 4/6 inhibitor (palbociclib, ribociclib, abemaciclib) | 8 | 9.5 |
| Immunotherapy (atezolizumab, pembrolizumab) | 0 | 0.0 |
| Denosumab | 11 | 13.1 |
| Bisphosphonate | 5 | 6.0 |
