# Supplementary material for: ESR1 F404 Mutations and Acquired Resistance to Fulvestrant in ESR1-Mutant Breast Cancer
Source: Cancer Discov. 2023 Nov 17;14(2):274–89. doi: 10.1158/2159-8290.CD-22-1387 (PMC10850945; doi:10.1158/2159-8290.CD-22-1387)
Supplement: Supplementary Figures 1-9 — Supplementary Figure 1. Progression free survival of patients with selected mutations. Supplementary Figure 2. H356Y does not activate estrogen signalling or alter fulvestrant sensitivity in combination with L536P. Supplementary Figure 3. Acquired ESR1 F404 mutations in patients treated with Fulvestrant. Supplementary Figure 4. Expression of individual ER target genes in RNAseq experiment. Supplementary Figure 5. Loss of F404L mutation in long-term culture of “E” mutant clones. Supplementary Figure 6. Response of D538G+F404L mutant models to elacestrant. Supplementary Figure 7. Response of D538G+F404L mutant models to camizestrant. Supplementary Figure 8. Response of D538G+F404L mutant models to 4OH tamoxifen. Supplementary Figure 9. Response of D538G+F404L mutant models to giredestrant. [file cd-22-1387_supplementary_figures_1-9_suppsf1-sf9.docx]

# Title: *ESR1* F404 mutations and acquired resistance to fulvestrant in *ESR1* mutant breast cancer.

## Authors and affiliations:

Belinda Kingston^1^, Alex Pearson^1^, Maria Teresa Herrera-Abreu^1^, Li-Xuan Sim^1^, Rosalind J Cutts^1^, Heena Shah^1^, Laura Moretti^2^, Lucy S Kilburn^2^, Hannah Johnson^2^, Iain R Macpherson^3^, Alistair Ring^4^, Judith M Bliss^2^, Yingwei Hou ^5^, Weiyi Toy^6^, John A Katzenellenbogen^5^, Sarat Chandarlapaty^6^, Nicholas C Turner^1,4^

^1^ The Breast Cancer Now Toby Robins Research Centre, The Institute of Cancer Research, London, SW3 6JB.

^2^ Clinical Trials and Statistics Unit at The Institute of Cancer Research, London, UK

^3^ School of Cancer Sciences, University of Glasgow, Glasgow, G61 1QH

^4^ Breast Unit, The Royal Marsden Hospital, Fulham Road, London.

^5^ Department of Chemistry and Cancer Center at Illinois, University of Illinois at Urbana-Champaign, Urbana, Illinois.

^6^ Memorial Sloan Kettering Cancer Center, New York City; Department of Medicine, Weill Cornell Medical College, New York City, USA.

## Running title:

Mutations of *ESR1* at F404 confer fulvestrant resistance.

## Keywords:

Fulvestrant, acquired resistance, breast cancer.


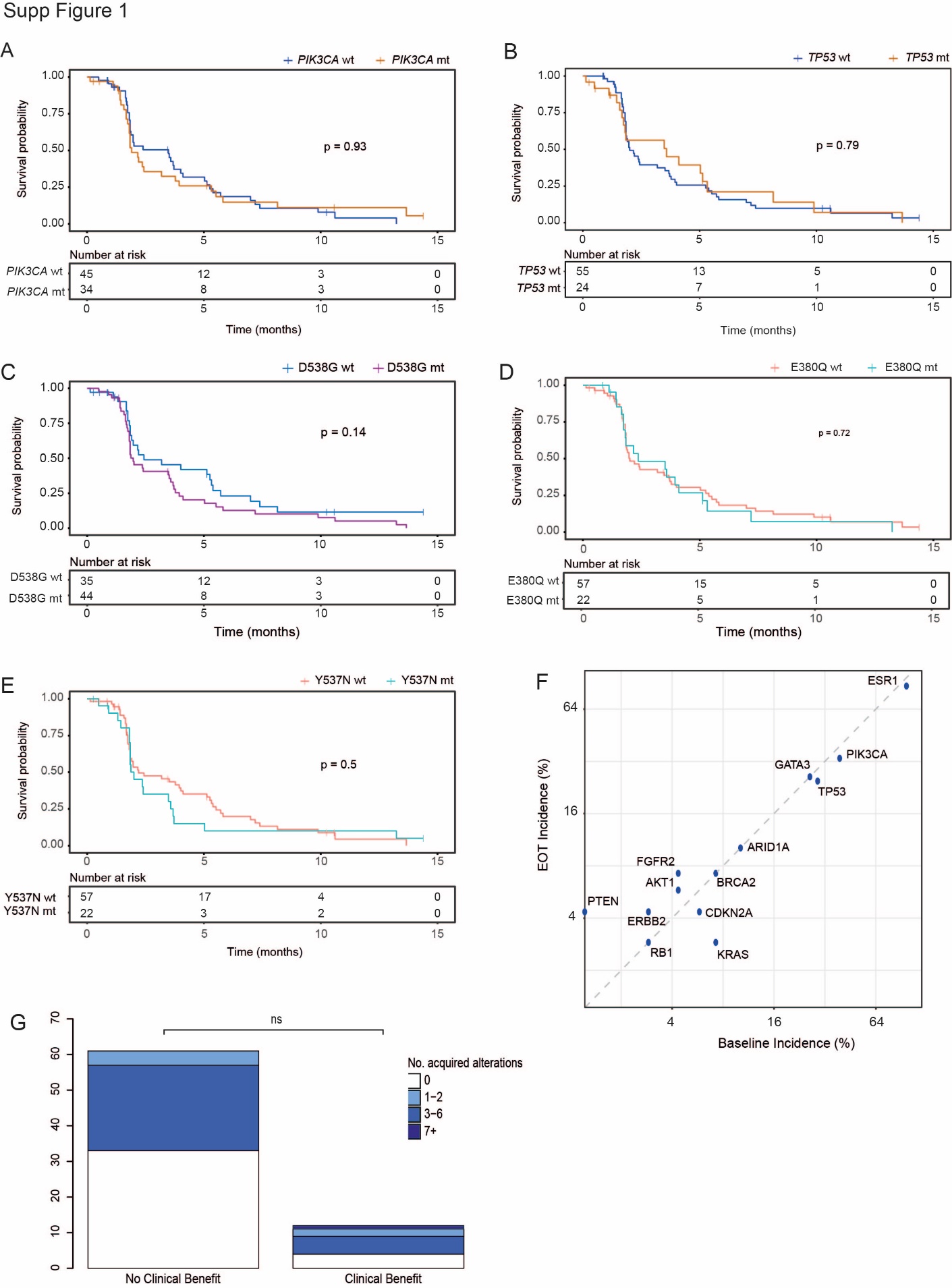


# Supplementary Figure 1. Progression free survival of patients with selected mutations.

A. Progression-free survival of patients in Cohort A with baseline *PIK3CA* mutations. p-value from log rank test. HR >1 denotes worse PFS for that group. HR 0.55, 95% CI 0.34 to 0.89. wt, wild type; mt, mutant.

B. Progression-free survival of patients in Cohort A with baseline *TP53* mutations. p-value from log rank test. HR >1 denotes worse PFS for that group. HR 1.79, 95% CI 1.04 to 3.07. wt, wild type; mt, mutant.

C. Progression-free survival of patients in Cohort A with baseline *ESR1* D538G mutations. p-value from log rank test. HR >1 denotes worse PFS for that group. HR 0.81, 95% CI 0.49 to 1.33. wt, wild type; mt, mutant.

D. Progression-free survival of patients in Cohort A with baseline *ESR1* E380Q mutations. p-value from log rank test. HR >1 denotes worse PFS for that group. HR 1.18, 95% CI 0.69 to 2.03. wt, wild type; mt, mutant.

E. Progression-free survival of patients in Cohort A with baseline *ESR1* Y537N mutations. p-value from log rank test. HR >1 denotes worse PFS for that group. HR 0.91, 95% CI 0.53 to 1.55. wt, wild type; mt, mutant.

F. Incidence of mutations in indicated genes at baseline vs end of treatment.

G. Number of acquired mutations in patients by clinical benefit (CR/PR/SD >= 24 weeks). Comparison by Chi-squared test.


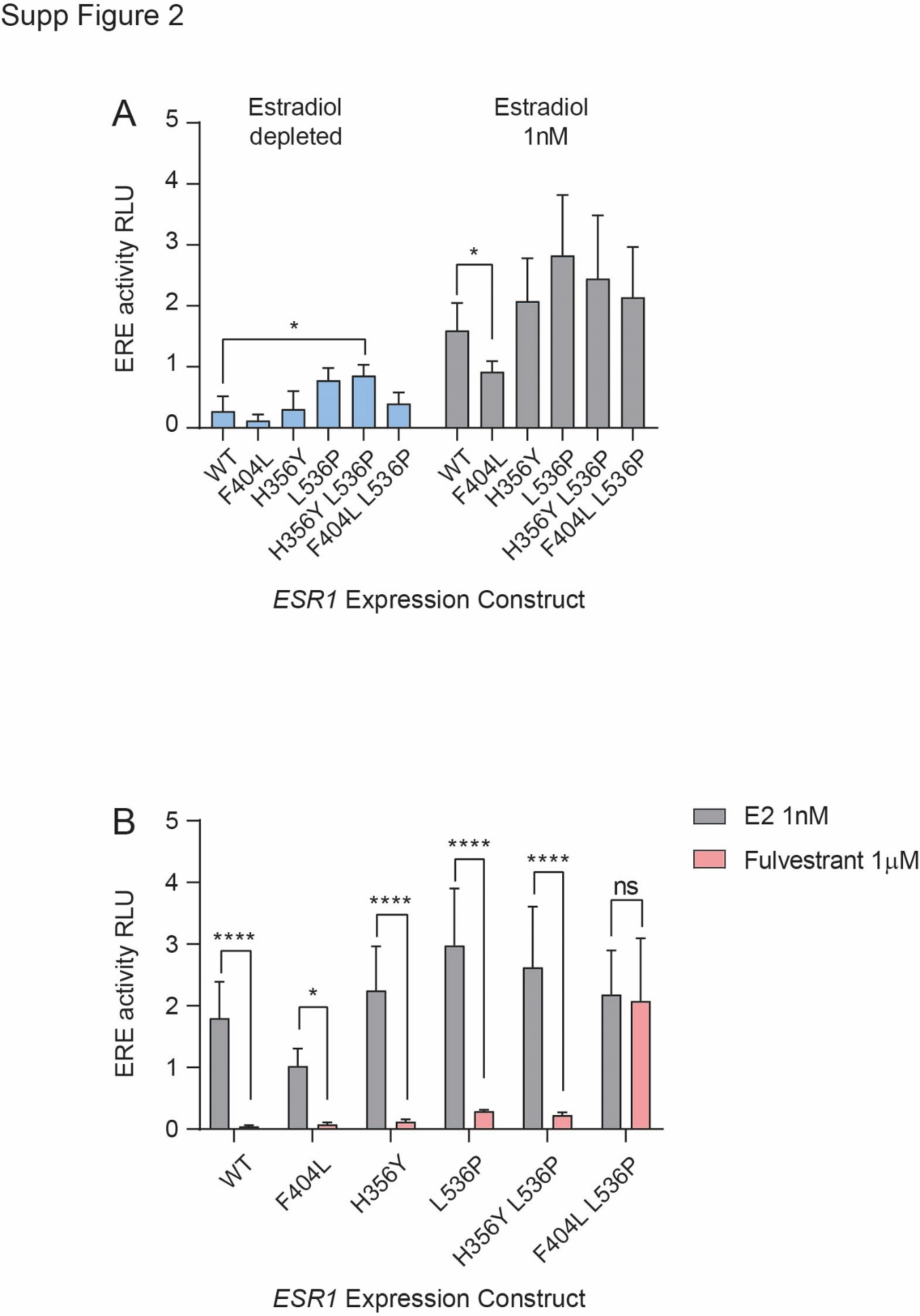


# Supplementary Figure 2. H356Y does not activate estrogen signalling or alter fulvestrant sensitivity in combination with L536P.

A. MCF7 cells were co-transfected with the indicated ESR1 expression constructs ERE-luciferase reporter and control construct. Cells were treated in either the absence or presence of estradiol (1nM) for 24 hours and ERE-luciferase activity assessed. 2-way repeated measures ANOVA with Dunnett’s multiple comparisons test, n=3 mean with SD, *P<0.05, **P<0.01, ****P<0.0001.

B. MCF7 cells were co-transfected with the indicated ESR1 expression constructs ERE-luciferase reporter and control construct. Cells were treated in either the absence or presence of fulvestrant (1µM) for 24 hours and ERE-luciferase activity assessed. 2-way repeated measures ANOVA with Sidak’s multiple comparisons test, n=3 mean with SD, *P<0.05, ***P<0.001, ****P<0.0001.

#
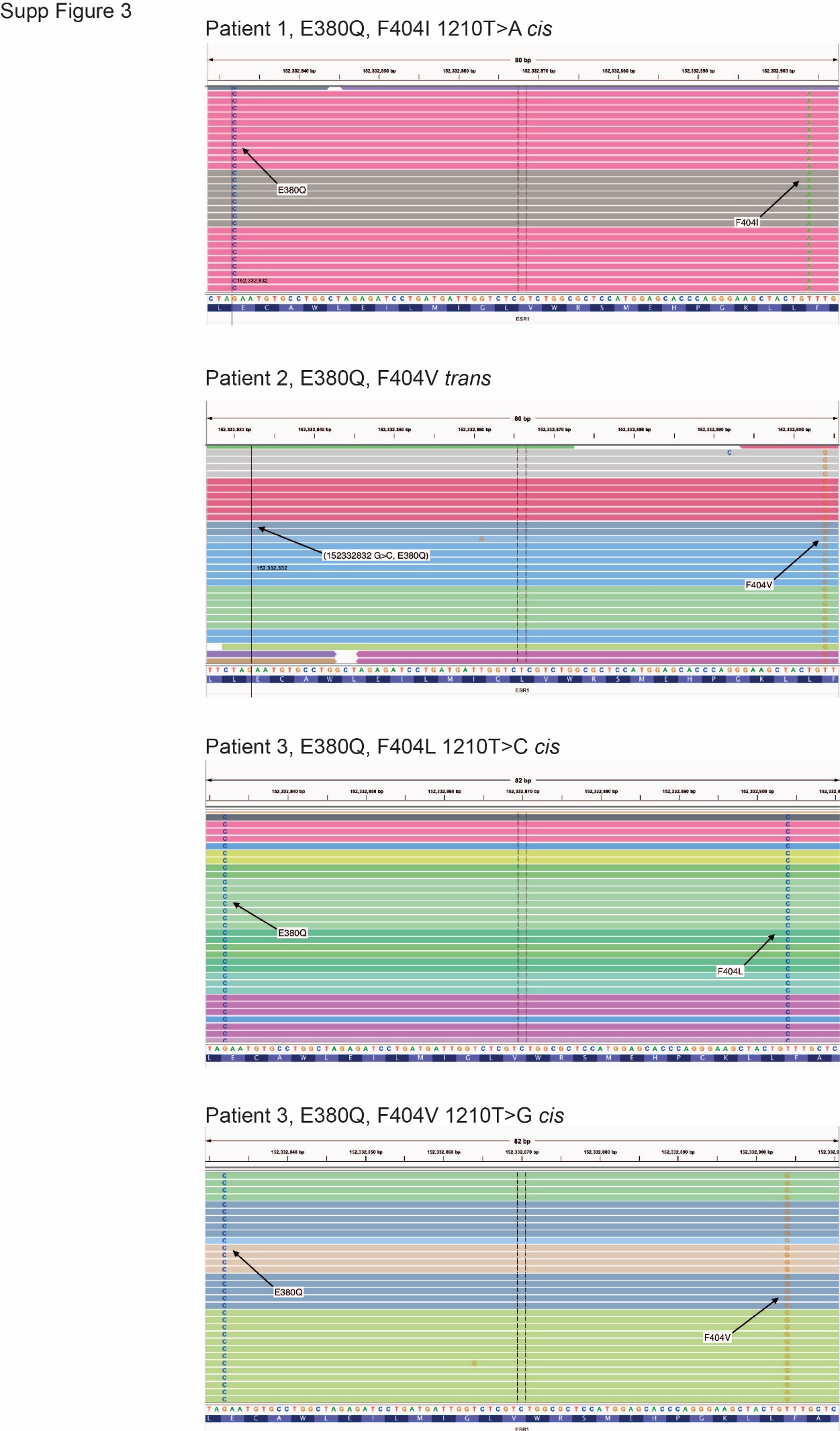
Supplementary Figure 3. Acquired *ESR1* F404 mutations in patients treated with Fulvestrant.

Individual reads visualised in Integrated Genome Viewer (IGV) (1-3)

#
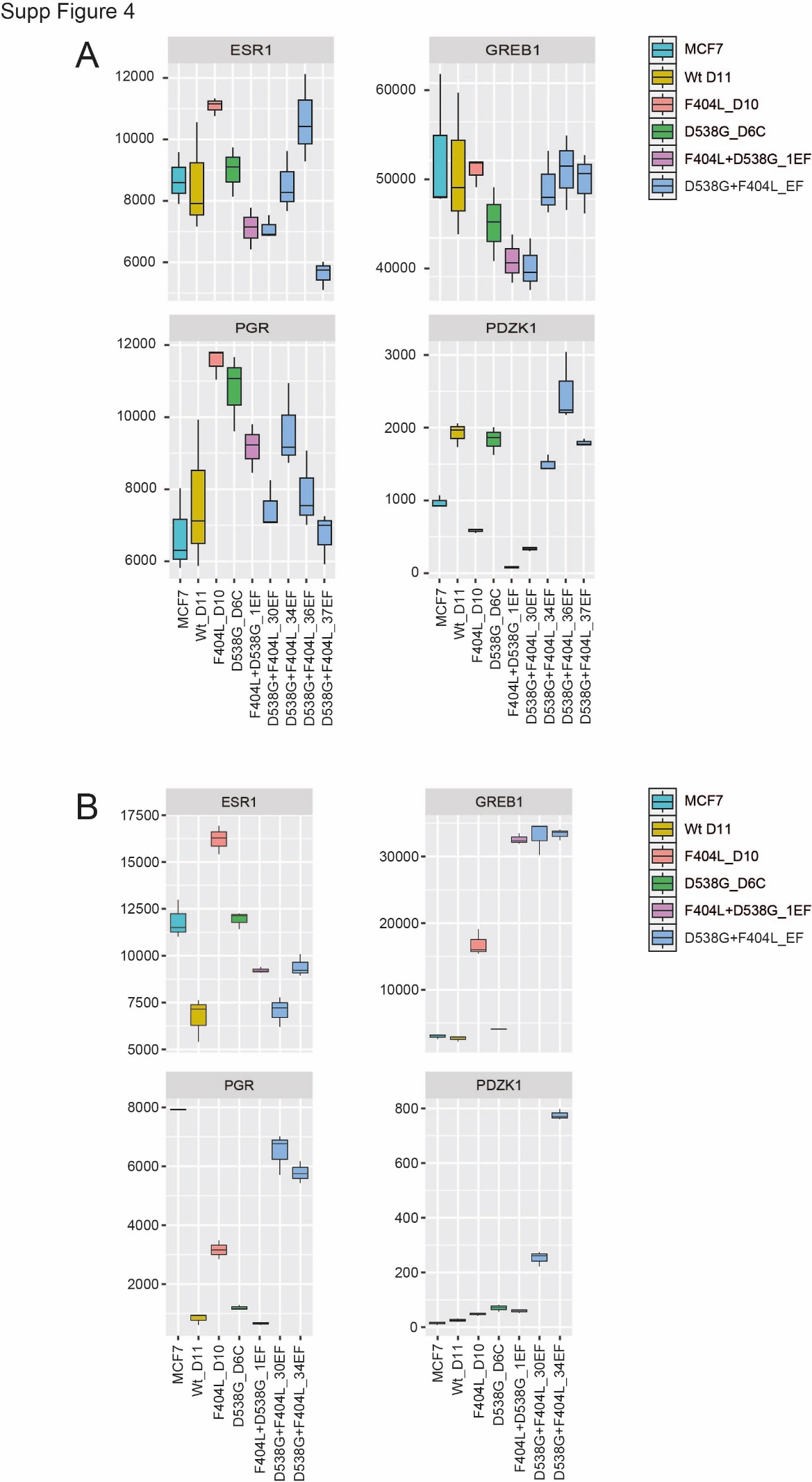
Supplementary Figure 4. Expression of individual ER target genes in RNAseq experiment

A. Read counts for selected estrogen response genes ESR1, GREB1, PDZK1 and PGR for the ESR1 mutant cell line models maintained in 1nM estradiol. Box represents the 25% and 75% interquartile range and whiskers represents counts data range.

B. Read counts for selected estrogen response genes ESR1, GREB1, PDZK1 and PGR for the ESR1 mutant cell line models maintained in 1mM Fulvestrant for 24hr. Box represents the 25% and 75% interquartile range and whiskers represents counts data range.


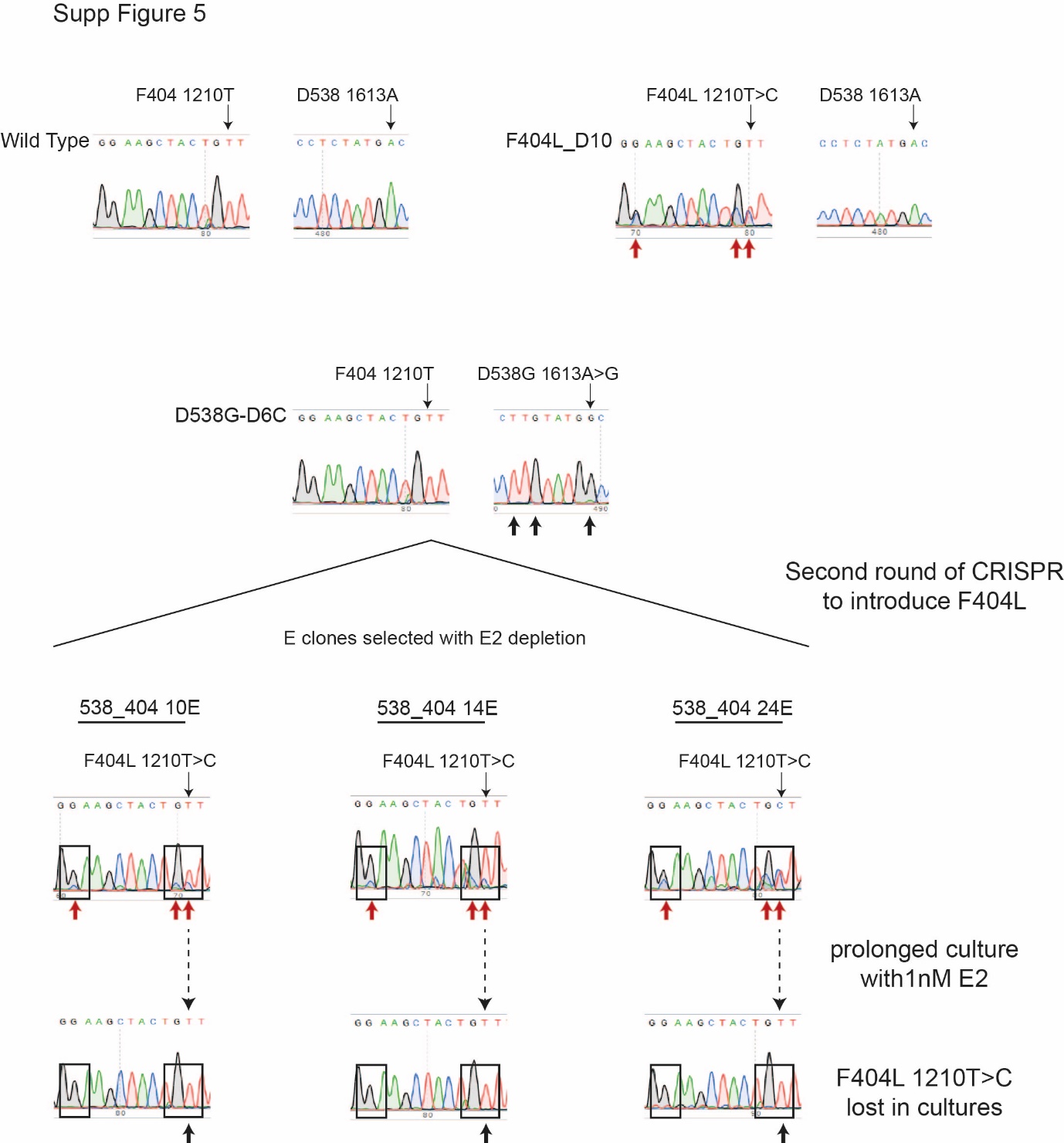


# Supplementary Figure 5. Loss of F404L mutation in long-term culture of “E” mutant clones.

CRISPR clones of MCF7 cells expressing *ESR1* F404L (1210T>C, CRISPR edit indicated by red arrows) or D538G (1613A>G; CRISPR edit indicated by black arrows) were identified by RT-PCR followed by Sanger sequencing (left hand panels). Each cell line has three mutations, the mutation introduced, and two additional silent mutations that destroy the CRISPR PAM sequence to prevent re-editing. A second round of CRISPR was used to introduce *ESR1* F404L (1210T>C) into a clone (D6C) that expressed D538G (1613A>G). Clones were selected in the absence of estradiol and expression of F404L confirmed by RT-PCR and Sanger sequencing. With prolonged culture F404L, and the PAM sequence changes, were observed to be lost from all three models.


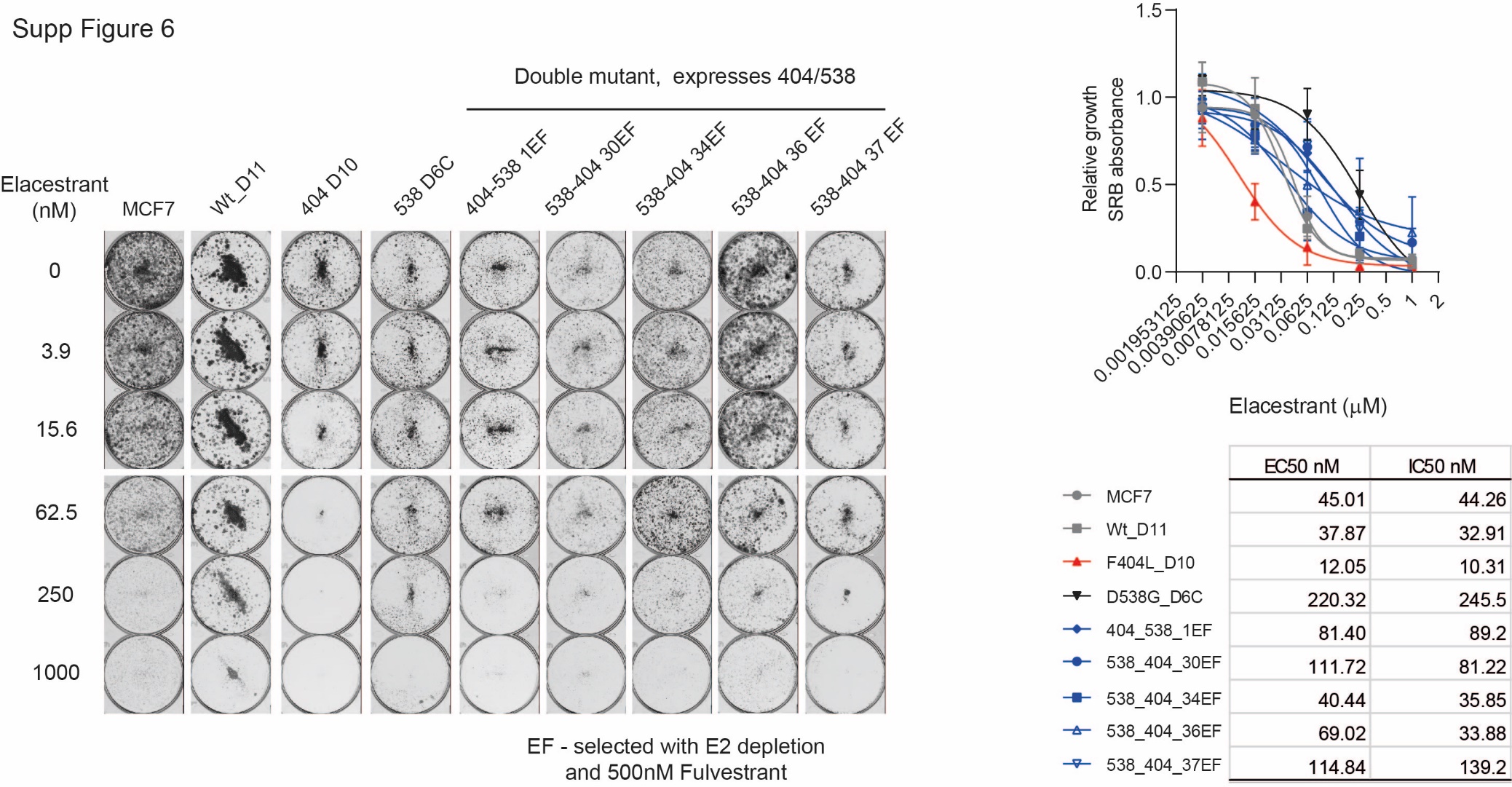


# Supplementary Figure 6. Response of D538G+F404L mutant models to elacestrant.

*Lefthand* panel, clonongenic assays grown in indicated concentrations of elacestrant for 14 days. *Righthand* panel, quantification of colony formation assays for *ESR1* mutant models. SRB stained colonies were dissolved and absorbance at 565nm measured. EC50 and IC50 values were calculated from the response curves. Mean with sem, n=3 independent experiments.


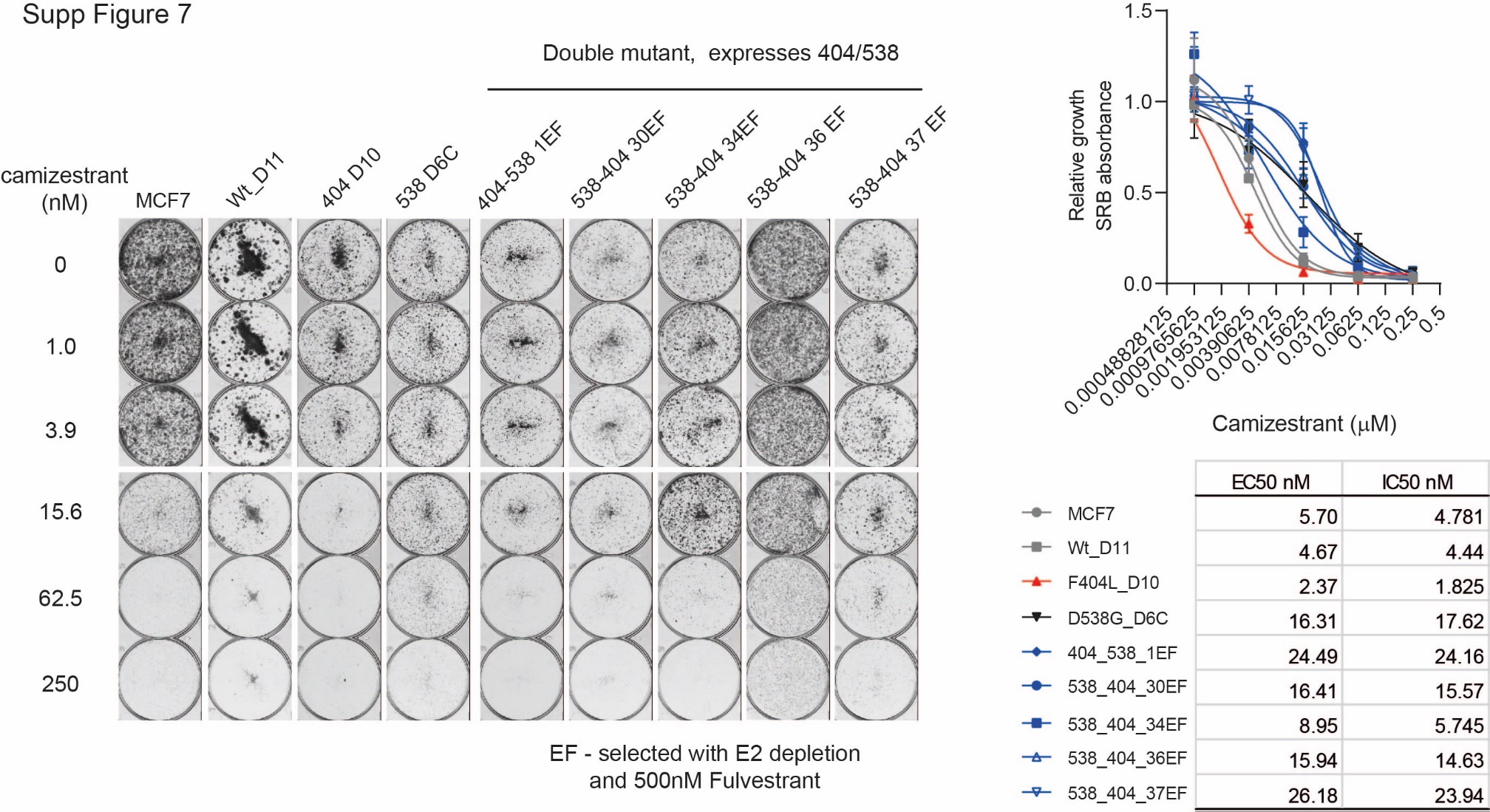


# Supplementary Figure 7. Response of D538G+F404L mutant models to camizestrant.

*Lefthand* panel, clonongenic assays grown in indicated concentrations of camizestrant for 14 days. *Righthand* panel, quantification of colony formation assays for *ESR1* mutant models. SRB stained colonies were dissolved and absorbance at 565nm measured. EC50 and IC50 values were calculated from the response curves. Mean with sem, n=3 independent experiments.


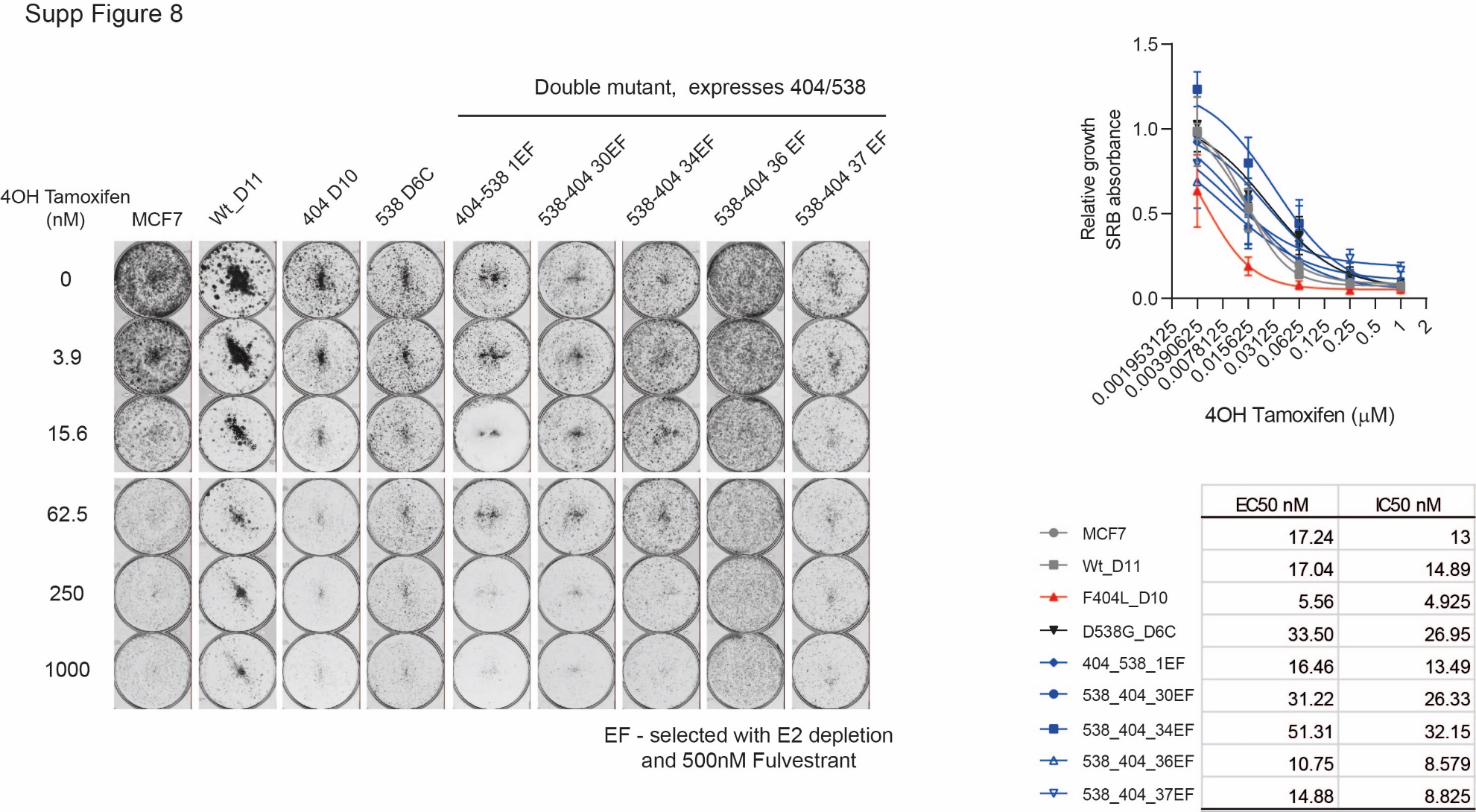


# Supplementary Figure 8. Response of D538G+F404L mutant models to 4OH tamoxifen.

*Lefthand* panel, clonongenic assays grown in indicated concentrations of 4OH tamoxifen for 14 days. *Righthand* panel, quantification of colony formation assays for *ESR1* mutant models. SRB stained colonies were dissolved and absorbance at 565nm measured. EC50 and IC50 values were calculated from the response curves. Mean with sem, n=3 independent experiments.


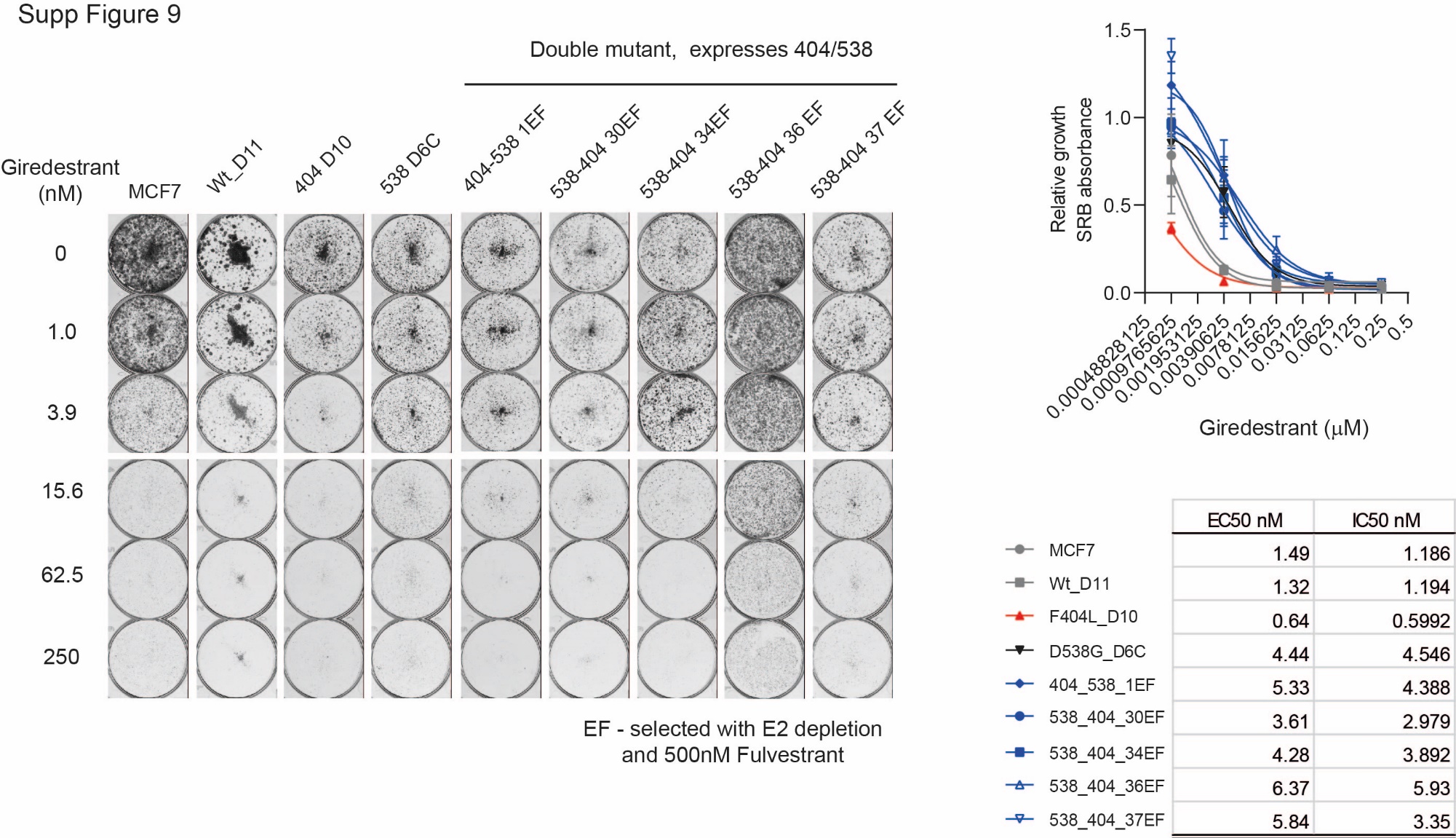


# Supplementary Figure 9. Response of D538G+F404L mutant models to giredestrant.

*Lefthand* panel, clonongenic assays grown in indicated concentrations of giredestrant for 14 days. *Righthand* panel, quantification of colony formation assays for *ESR1* mutant models. SRB stained colonies were dissolved and absorbance at 565nm measured. EC50 and IC50 values were calculated from the response curves. Mean with sem, n=3 independent experiments.
